# The Development of Visible-Light Organic Photocatalysts for Atom Transfer Radical Polymerization via Conjugation Extension

**DOI:** 10.3390/molecules29122763

**Published:** 2024-06-11

**Authors:** Hui Shao, Runzhi Long, Hui Xu, Pan Sun, Guangrong Wang, Yuanming Li, Saihu Liao

**Affiliations:** 1 Key Laboratory of Molecule Synthesis and Function Discovery (Fujian Province University), College of Chemistry, Fuzhou University, Fuzhou 350108, China221320378@fzu.edu.cn (H.X.);; 2 State Key Laboratory of Physical Chemistry of Solid Surfaces, College of Chemistry and Chemical Engineering, Xiamen University, Xiamen 361005, China

**Keywords:** ATRP, visible light, extended conjugation, organocatalysis, photocatalysts

## Abstract

This work aimed to develop organic photocatalysts (PCs) that could mediate organocatalytic atom transfer radical polymerization (O-ATRP) under visible light. Through the core-modification of known chromophoric structures and ring-locking to reach a conjugation extension, annulated *N*-aryl benzo[kl]acridines were identified as effective visible light-responsive photocatalysts. The corresponding selenium-doped structure showed excellent performance in the O-ATRP of methacrylates, which could afford polymer products with controlled molecular weights and low dispersities under the irradiation of visible light at a 100 ppm catalyst loading.

## 1. Introduction

Reversible deactivating radical polymerization (RDRP) is a polymerization technology that has been rapidly developing in the past three decades and has found wide applications in many fields [1,2,3]. The application of light regulation in RDRP-based polymer synthesis has been an emerging and rapidly growing research area over the last ten years [4]. The advantage of light as an external stimulus to regulate the polymerization process includes the non-invasive mediation, high energy efficiency, spatiotemporal control, wide availability, and easy access to inexpensive, long-wavelength LED light sources [3]. In particular, the corresponding visible light-mediated RDRP offers photocontrol technologies enabling temporal and spatial regulation of chain growth, a capability not attainable in conventional thermally activated and chemically activated processes [4,5]. As one of the most powerful and versatile RDRP techniques, atom transfer radical polymerization (ATRP) [6,7] has gained significant advances in the past years by uniting light regulation, and the corresponding photocontrolled ATRP (photo-ATRP) routes not only allow for the precise control of reaction kinetics but also facilitate the fabrication of advanced materials [8,9,10].

**Figure 1:**
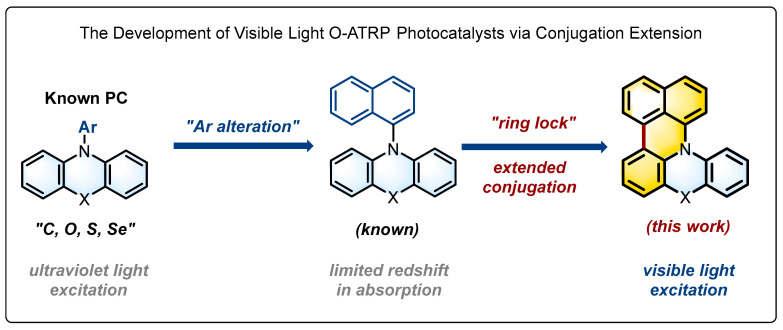
The development of visible-light O-ATRP photocatalysts with extended conjugation.

Light-mediated radical polymerization has profited from the rapid development of organic photocatalysts (PCs). In this regard, the development of photocatalysts derived from phenothiazine [11,12], phenazine [13], and phenoxazine [14,15] core structures (Figure 1, left) with favorable redox balance between reducing excited states and oxidizing radical cations to mediate the activation–deactivation equilibrium [16] has significantly pushed forward the field of O-ATRP. To further increase the performance of these photocatalysts, various modifications have been introduced to the core structures, aiming to enhance their absorption of visible light [16] and enable O-ATRP with good control under longer wavelength light rather than ultraviolet [17,18,19,20,21]. On the other hand, variation in the heteroatoms in these structures has also been investigated, including systematic studies on the effect of chalcogenide atoms [22,23], which not only improved our understanding of the structure–property relationships but also offered a tentative guideline for tailoring the structure of known PCs.

Along with the modifications of known chromophoric structures like phenothiazine, our research interest has moved toward developing photocatalysts with distinct parental skeletons. In this regard, we became particularly interested in the polycyclic arenes with a conjugated structure larger than that of anthracene as a parental framework for “doping”, following the design logic of heteroatom doping of polycyclic arenes for the development of new photocatalyst [23,24,25,26]. Herein, we wish to report that, following additional structural refinement, *N*-naphthyl phenochalcogenazine analogue (Figure 1, middle) could evolve into a visible-light photocatalyst, with its absorption maximum redshifting to above 400 nm after “locking” the naphthyl group, which could thus serve as a visible-light photocatalyst for the O-ATRP of methacrylates. These locked structures possess an extended conjugation (Figure 1, right), with the benzo[kl]acridine moiety (highlighted in yellow) exhibiting a strong absorption of purple-blue light. Upon further variation in the doping atoms (X = C, O, S, and Se), the corresponding selenium-doped structure was found showing the best performance in the O-ATRP of methacrylates under the irradiation of visible light, allowing for the synthesis of polymers with controlled molecular weights and low dispersities.

## 2. Results and Discussion

### 2.1. Photocatalyst Development

**Molecular Design and Synthesis.** To increase the visible-light absorption of the phenothiazine and phenoxazine chromophoric structures, core-modification via alteration of the Ar group (e.g., replacing the phenyl group with a naphthyl group) has been commonly employed, but the improvement like redshift of absorption was often limited [27,28]. To generate a structure with an extended conjugation, we conceived the combination of Ar alteration with “ring lock” could lead to a new organic photocatalyst for O-ATRP that could probably be effective under visible light (Figure 1). We thus synthesized five photocatalysts with a benzo[kl]acridine moiety, by varying the exo-doping atoms (X = C, O, S, Se, shown below). The detailed synthesis is described in the Supporting Information. These structures can be readily prepared from phenochalcogenazines through sequential N–H/C–H coupling with 1,8-dibromonaphthalene in a sequential transformation [29,30,31].

**Figure 2:**
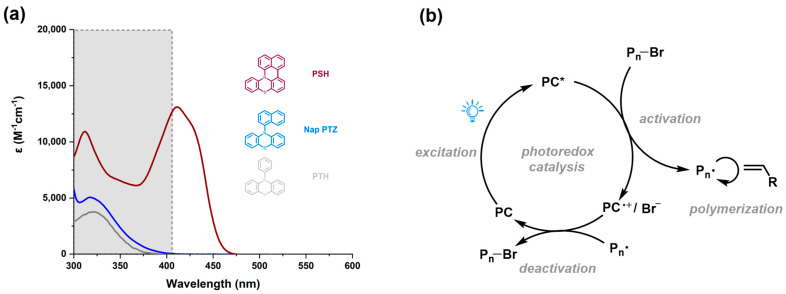
(**a**) A comparison of UV–Vis absorption profiles of **PSH**, **Nap-PTZ**, and **PTH**. (**b**) A typical mechanism for the photoredox catalyst-mediated O-ATRP.

**Properties of the catalysts:** The UV-Vis absorbance of each PC was first measured, and the results are summarized in Table 1. The corresponding UV-Vis absorption spectra are in Supplementary Figure S12. All five photocatalysts exhibited a strong absorption in the range of 350–450 nm, with the maximum absorption wavelength located at 408–435 nm. A comparison of the absorption profiles of **PSH** with known photocatalyst *N*-naphthyl phenothiazine (**Nap-PTZ**) and *N*-phenyl phenothiazine (**PTH**) is depicted in Figure 2a. Replacing the *N*-phenyl group in **PTH** with a larger aryl group like naphthyl was commonly used to enhance the catalysts’ absorption of visible light, but the improvement was often limited. In contrast, the absorption profile of **PSH** was not only redshifted (Δλ_max_ = 95 nm versus **Nap-PTZ**) into the visible-light region (λ_max_ = 412 nm), and it also exhibited a significantly enhanced molar extinction coefficient (ε = 13,111 M^−1^cm^−1^ at λ_max_ = 412 nm), making it more efficient in absorbing visible light than the unlocked structure (**Nap-PTZ**). In fact, all five photocatalysts exhibited a strong visible-light absorption with molar extinction coefficients of ε_max_ > 10,000 M^−1^ cm^−1^, which could favor their excitation by longer wavelength light during the photocatalytic polymerization (Figure 2b) and thus decrease the catalyst loadings [32,33].

Cyclic voltammogram (CV) measurements were also performed with these catalysts, and all the five catalysts, including **PSH** and **PSeH**, showed a reversible CV, suggesting the corresponding radical cations’ good stability, which is critical in the deactivation step to convert the propagating radicals (P_n_**·**) into dormant chains. Based on the *E*_1/2_(PC^•+^/PC) values obtained from CV measurements and the λ_em_ of fluorescence emission, the reduction power of the excited states (^1^PC*) of these catalysts can be calculated. As shown in Table 1, all of these catalysts possess a good reducing ability at the excited states (−2.00 to −2.25 V vs. SCE) that is enough to reduce the alkyl bromide initiators via single-electron transfer [11,12]. Moreover, DFT calculations were performed to evaluate the reduction capability of the triplet states [34,35,36,37,38], and the *E*^0^* values (PC^•+^/^3^PC*) are summarized in Table 1, in the range from −1.43 to −1.78 V vs. SCE. The *E*^0^* values (PC^•+^/^3^PC*) obtained with our method for **PCH** and **PSeH** (−1.78 V and −2.04 V vs. SCE) show good agreement with the experimental values (−2.25 V and −2.03 V vs. SCE). In addition, **both** *Eº*_exp_(PC^•+^/^1^PC*) **and** *Eº*_theo_(PC^•+^/^3^PC^*^) follow similar trends to those seen in the excited-state energies (i.e., decreasing in magnitude from **POH** to **PSH**, and then increasing from **PSH** to **PSeH**) (Supplementary Table S1). Furthermore, heavy-atom (e.g., Se) doping can often enhance the spin–orbit coupling (SOC) and favor the intersystem crossing from singlet states into triplet states, which could often improve the initiation and activation in O-ATRP [39,40].

**Figure 3:**
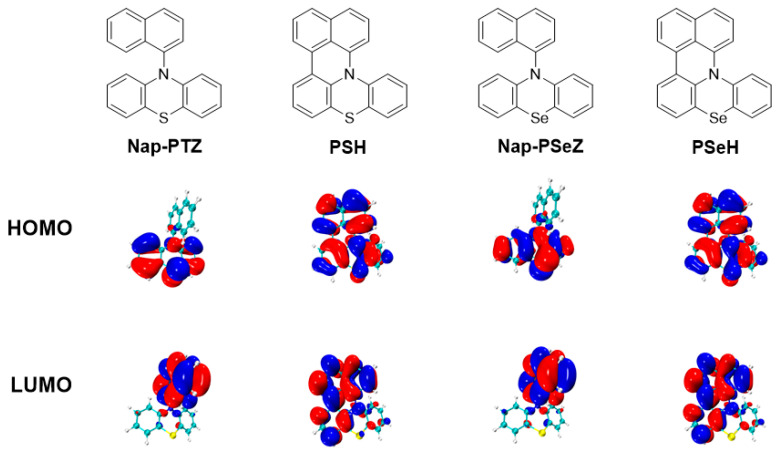
The HOMO and LUMO distribution of **Nap-PTZ**, **PSH**, **Nap-PSeZ**, and **PSeH**.

On the basis of time-dependent density functional theory (TD-DFT) calculations at the B3LYP/6-311+G (d)/LANL2DZ level of theory, the HOMOs and LUMOs are mainly located on the donor and acceptor groups, respectively (HOMO = highest occupied molecular orbital; LUMO = lowest unoccupied molecular orbital) (Figure 3). Among these PC molecules, **PSH** and **PTZ** are similar in that they have one phenothiazine donor attached to the naphthalene ring, but with a different conjugation due to the lock or not of the naphthalene ring. Comparing **PSH** and **PTZ**, we see that the locked structure has a large contribution to both HOMO and LUMO density, and the HOMO and LUMO overlap is extensive when the naphthalene ring is locked. Therefore, the molecular orbital picture of **PSH** suggests an obvious expanded conjugation. Furthermore, an examination of the triplet state (^3^PC*) frontier orbitals also reveals differences between locked-structure PCs and the unlocked ones (Supplementary Figure S17). The low-lying singly occupied molecular orbital (SOMO) of the **PSeH** differs from **Nap-PSeZ**, of which the electron is mainly localized over the phenothiazine π system. This molecular orbital picture also indicates an obvious extended conjugation in these locked structures.

### 2.2. Catalyst Evaluation

The catalytic performance of PCs was initially evaluated in the polymerization of methyl methacrylate (MMA) in dichloromethane (DCM) by using diethyl 2-bromo-2-methylmalonate (DBMM) as an initiator at a [MMA]_0_/[DBMM]_0_/[PC]_0_ ratio of 1000/10/0.1. As shown in Table 2, **PNH** afforded a poly(MMA) product with a high *Đ* (1.49) and a number average molecular weight that deviates significantly from the corresponding theoretical values (entry 1). **POH** exhibited a better catalytic performance in the O-ATRP of MMA when compared to **PNH**, producing PMMA with a similar dispersity but a much higher initiator efficiency (I* = 93%, entry 2). The introduction of the S atom also gave a higher I* (74%, entry 4) in comparison to **PNH** (entry 1) and **PCH** (entry 3). Entries 5 and 6 in Table 2 are the results of polymerizations under ultraviolet (UV) light for 8 h. In this case, **PSH** could also afford MMA polymer products with lower dispersity. To our delight, among these catalysts, the corresponding selenium analogue (**PSeH**) showed the best performance in terms of molecular-weight control (I* > 85%) and low dispersity (*Đ* 1.24 at >80% conversion), and both purple and white LEDs are suitable to achieve a controlled radical polymerization (entries 7 and 8). In contrast, the unlocked one (**Nap-PSeZ**) gave a less controlled polymerization and a much lower initiator efficiency (entry 9).

### 2.3. Kinetic Study, Temporal Control, and Polymerization Extension

**Figure 4:**
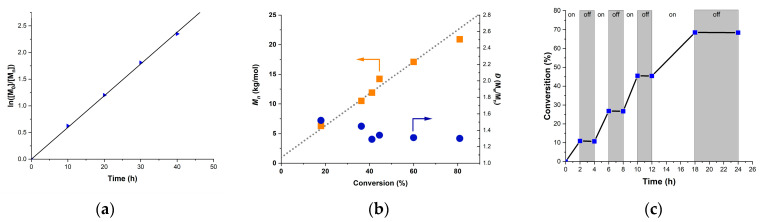
Kinetic plot for the **PSeH**-catalyzed metal-free ATRP of MMA. (**a**) Plot of ln([M_0_]/[M_t_]) as a function of reaction time. (**b**) Plot of Mn and *Đ* versus monomer conversion. (**c**) Light on–off experiment. Polymerization conditions: [MMA]_0_:[DBMM]_0_:[**PSeH**]_0_ = [2000]:[10]:[0.2] in DCM, under the irradiation of white LEDs.

The kinetics of the polymerization of MMA were studied at catalyst loadings of 100 ppm, as shown in Figure 4a,b, which unveiled a linear growth of molecular mass upon monomer conversion (up to 80% conversion). It can be observed that there is a linear relationship in the whole process of polymerization, and the dispersity remains within a relatively narrow range, demonstrating a constant concentration of propagating radicals and a pseudo first-order kinetics of the photo-ATRP. Effective temporal control over the polymerization by irradiation was also demonstrated through the light on–off experiment (Figure 4c), and no monomer conversion was observed during the dark periods. It can be assumed that photoexcited PCs activate the dormant species, and the chain grows only when the light is on, thus achieving a light regulation over the chain propagation.

**Figure 5:**
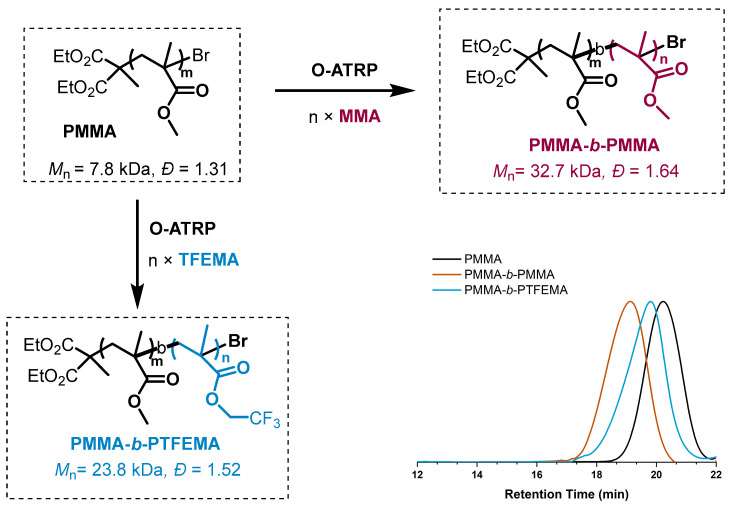
Chain extension with **PSeH** and block copolymer synthesis from PMMA macroinitiator.

**PSeH** was further applied to the polymerization of different monomers, and the results are summarized in Table 3. The polymerization of methacrylate monomers, including benzyl methacrylate (BzMA), trifluoroethyl methacrylate (TFEMA), tert-butyl methacrylate (*t*-BMA), and 3-(trimethoxysilyl)propyl methacrylate (TSPMA), proceeded in a controlled manner in terms of molecular weight and low dispersity under white LEDs (entries 1–5). It is worth noting that the **PSeH** could also deliver an efficient polymerization of glycerol acrylate (GMA). Furthermore, chain-extension polymerizations were also carried out using the isolated PMMA macroinitiator (*M*_n_ = 7.8, *Đ* = 1.31), which was prepared with **PSeH** as the photocatalyst and DBMM as the initiator under the standard reaction conditions. As shown in Figure 5, two block polymers, PMMA-*b*-PMMA and PMMA-*b*-PTEFMA, were successfully prepared, indicating that good fidelity of the chain-end groups was achieved in the **PSeH**-mediated radical polymerization.

## 3. Materials and Methods

### 3.1. Materials and Instruments

The monomers (methyl methacrylate (MMA), 2,2,2-trifluoroethyl methacrylate (TFEMA), 3-(trimethoxysilyl)propyl methacrylate (TMSPA), benzyl acrylate (BzA), and glycidyl methacrylate (GMA)) were dried over calcium hydride, distilled, and stored under an inert atmosphere at 2–8 °C. Diethyl 2-bromo-2-methylmalonate (DBMM) was purchased from TCI Chemicals and dried over calcium hydride, distilled, and stored under an inert atmosphere at 2–8 °C. Dichloromethane (DCM), tetrahydrofuran (THF), and toluene were dried over calcium hydride, distilled, and stored under an inert atmosphere at 2–8 °C. Other solvents, *N*,*N*-dimethylacetamide (DMAc), *N*,*N*-dimethylformamide (DMF), and ethyl acetate (EtOAc), were purchased from J&K Seal and used as received.

^1^H NMR and ^13^C NMR spectra were recorded on a Bruker AVIII 400 or a Bruker Avance 500 spectrometer (Bruker, Billerica, MA, USA), using dimethylsulfuxide-d_6_ (DMSO-d_6_) or CDCl_3_ as the solvent. Analysis of polymer samples was performed via gel permeation chromatography (GPC), using an Agilent HPLC (Agilent Technologies Inc., Santa Clara, CA, USA), two Shodex GPC KD-806M gel permeation columns (8.0 mm ID × 300 mm), and a Wyatt Technology TrEX differential refractometer (Wyatt Technology, Santa Barbara, CA, USA), using THF as the eluent at a flow rate of 1.0 mL/min. The GPC system was calibrated based on narrow molecular distributions of poly(methyl methacrylate) standards with molecular weights between 602 and 2,200,000 g·mol^−1^. Cyclic voltammogram (CV) measurement experiment was carried out with a CHI660 D electrochemical workstation (Shanghai Chenhua Instrument Plant, Shanghai, China), with 0.1 mol of tetrabutylammonium hexafluorophosphate as the electrolyte at room temperature, and an Ag/AgCl electrode was used as the reference electrode. UV/Vis/NIR spectra were recorded on a Perkins Elmer Lambda 900 spectrometer (PerkinElmer Life and Analytical Sciences, Shelton, CT, USA) equipped with a PTP-1 Peltier temperature controller, and steady-state emission spectra were acquired using Edinburgh Instruments, FLS980 spectrometer (Edinburgh Instruments, Livingston, UK). Mass spectra were recorded on an Agilent Q-TOF 6520 system (Agilent Technologies Inc., Santa Clara, CA, USA) using electrospray ionization in Positive/Negative ion detection (ESI^+^/ESI^−^) mode, and the significant fragments are reported in the following fashion: *m*/*z* (relative intensity).

### 3.2. Synthesis and Characterization of Catalyst **PSeH**

Synthesis of **PSeH** precursor **RM 5**: Bis (2-iodophenyl) amine (1.26 g, 3 mmol, 1 eq.), Se powder (0.474 g, 6 mmol, 2eq.), and KOH (0.672 g, 12 mmol, 4 eq.) were dissolved in 30 mL of DMSO. The reaction mixture was stirred at 110 °C for 24 h. At room temperature, the reaction mixture was diluted with saturated NH_4_Cl aq. solution and DCM. The aqueous phase was extracted by DCM three times and dried with Na_2_SO_4_, and the solvent was removed by rotary evaporation. The crude product was purified by flash chromatography, with a yield of 75%.

Then, 1,8-dibromonaphthalene (3.5 mmol, 1 g), the abovementioned obtained precursor **RM 5** (3 mmol), NaO*^t^*Bu (7 mmol, 0.67 g), Pd (OAc)_2_ (3 mol%, 0.09 mmol, 0.02 g), Pd_2_(dba)_3_ (3 mol%, 0.09 mmol, 0.082 g), Cy_3_P (7 mol%, 0.21 mmol, 0.06 g), and (*t*Bu)_3_P (7 mol%, 0.21mmol, 0.043 g) were stirred in 50 mL of dried toluene at 90 °C for 10 h. Then, the solvent was removed under reduced pressure, and the residue was purified by column chromatography, using hexane/dichloromethane as eluent on silica. Finally, the target products were obtained in 45–78% yield. Further recrystallization was conducted using hexane and DCM. **PSeH** ^1^H NMR ((500 MHz, CDCl_3_) *δ* 7.73 (t, *J* = 7.6 Hz, 2H), 7.63 (d, *J* = 8.2 Hz, 1H), 7.49–7.44 (m, 1H), 7.40 (d, *J* = 8.2 Hz, 2H), 7.35–7.30 (m, 2H), 7.21 (d, *J* = 7.6 Hz, 1H), 7.13–7.07 (m, 2H), 7.05 (td, *J* = 7.5, 1.0 Hz, 2H).) **^13^C NMR** (126 MHz, CDCl_3_) δ 144.4, 140.4, 138.1, 134.2, 131.3, 129.8, 129.2, 127.9, 127.2, 126.6, 126.2, 125.9, 125.3, 125.0, 124.3, 122.5, 121.5, 120.7, 120.4, 116.2, 114.1. **HRMS (*m*/*z*):** calcd for [M + H]^+^ C_22_H_14_NSe, 372.0286; found 372.0271. For characterization (NMR spectrum, UV-Vis, emission spectrum, lifetimes, and CV spectrum), please see the Supporting Information.

### 3.3. Typical Procedures for Photoinduced O-ATRP

A typical O-ATRP procedure under the standard reaction conditions is as follows: in the glovebox, MMA (0.5 mL, 4.7 mmol, 100 eq.) as the model monomer, DBMM (9 μL, 47 μmol, 1 eq.), **PSeH** (0.35 mg, 0.94 μmol, 0.02 eq), and 0.5 mL of DCM were successively added to the Schlenk tube. Subsequently, the tube was placed under the irradiation of purple or white LEDs for a specified reaction time. Then, the tube was opened under argon, and a 20 μL of reaction mixture was syringed out and added to CDCl_3_ (containing 250 ppm BHT) for ^1^H NMR analysis to determine the monomer conversion. After polymerization, the reaction mixture was poured into methanol (30 mL), and the precipitates were collected and dried to afford the purified polymer for further analysis by GPC.

### 3.4. Polymerization Procedure for Chain Extension from PMMA Macroinitiator

MMA (2.00 mL, 18.8 mmol, 100 eq.), DBMM (72 μL, 283 μmol, 1.5 eq.), and catalyst (0.188 μmol, 0.01 eq.) were dissolved in 2.50 mL DCM and reacted according to the above general polymerization procedure for 16 h. After that, the tube was opened under argon, and 20.0 μL of the mixture was syringed out and quenched into CDCl_3_ containing 250 ppm BHT to determine the monomer conversion by ^1^H NMR (Conv. = 70.4%). At this time, the reaction mixture was poured into 250 mL methanol and stirred for 4 h. The resulting precipitate was slowly dripped into room-temperature methanol, after stirring for half an hour, and then isolated by vacuum filtration and washed with excess methanol. The polymer was then re-dissolved in a minimal amount of DCM again and dripped into 150 mL of methanol and stirred for 2 h to fully remove unreacted monomer, initiator, or catalyst. The resulting PMMA was collected via vacuum filtration and dried in a vacuum, at 40 °C, overnight, and obtained 1g of polymer, Mn = 7.8 kDa, *Ɖ* = 1.31.

**PMMA-*b*-PMMA:** A Schlenk tube with a PTFE stirring bar was charged with 0.35 mg of **PSeH** (9.42 × 10^−4^ mol, 0.1 eq.) and 74 mg of the PMMA macroinitiator described above (*M*_n_ = 7.8 kDa, 1.0 eq.) that was dissolved in 1 mL of DCM. Then, 200 μL of MMA was added (0.19 mmol, 200 eq.) and reacted according to the above general polymerization procedure for 16 h. Then, the reaction mixture was loaded into a syringe and slowly dripped into room-temperature methanol to precipitate the polymer. The product was re-dissolved in HPLC-grade, unstabilized tetrahydrofuran, and the Mn and *Ɖ* were analyzed by GPC.

**PMMA-*b*-PTFEMA:** A Schlenk tube with a PTFE stirring bar was charged with 50 μL of PC (9.42 × 10^−4^ mol, 0.05 eq.) and 74 mg of the PMMA macroinitiator described above (*M*n = 7.8 kDa, 1.0 eq.) which were dissolved in 1 mL of DCM. Then, 132 μL of TFEMA was added (1.12 mmol, 124 eq.) and reacted according to the above general polymerization procedure for 9 h. Then the reaction mixture as loaded into a syringe and slowly dripped into room temperature methanol to precipitate the polymer, The product was re-dissolved in HPLC grade, unstabilized tetrahydrofuran and the *M*_n_ and *Ɖ* were analyzed by GPC.

### 3.5. General Methods for Analysis of Kinetics and Molecular Weight Growth

A typical procedure of kinetics experiments were performed in glovebox, using a [MMA]:[DBMM] ratio of 200:1, with 100 ppm **PSeH** and 1 mL:1.5 mL MMA:DCM. To evaluate the kinetics and growth of molecular weight versus conversion for polymerization, an aliquot of 0.05 mL of reaction mixture was taken and injected into a solution of CDCl_3_ containing 250 ppm of the radical inhibitor (BHT) at predetermined times after the start of the polymerization, as indicated (when the reaction mixture was exposed to light). After NMR analysis, the sample was subjected to GPC analysis to determine the *M*_n_ and *M*_w_/*M*_n_ values.

## 4. Conclusions

Based on the idea of “locking” the *N*-naphthyl group in the *N*-naphthyl phenothiazine, a series of new photocatalysts with extended conjugation were forged which exhibited strong absorption in the visible-light region (λ_max_ = 412 nm), with a significant redshift (Δλ_max_ = 95 nm) from ultraviolet to purple-blue light. This distinct feature, in combination with suitable redox potentials, allows the corresponding O-, S-, and Se-doped photocatalysts to mediate the atom transfer radical polymerization of MMA under the irradiation of visible light rather than UV light. Notably, the selenium photocatalyst (**PSeH**) showed an excellent performance and a high initiating efficiency, which could deliver the polymer products with well-controlled molecular weights and low dispersities (*Đ* as low as 1.24) at a 100 ppm catalyst loading only. Furthermore, chain-extension and light on/off experiments were also performed, suggesting a good chain-end fidelity and good temporal control over the chain growth. In conclusion, the conjugation extension is demonstrated as a viable approach and also important for designing/modifying photocatalysts for O-ATRP, with the aim to improve the absorption of visible light and catalytic activity. We anticipate that these new photocatalysts could find further application in both small-molecule and macromolecule synthesis in the future.

## Figures and Tables

**Table 1:** Photophysical and electrochemical properties of PCs.

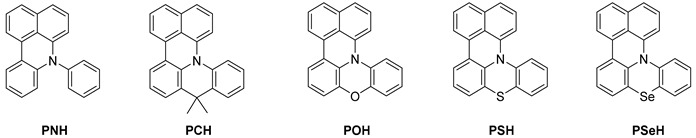									
PC	λ_max_ (nm) ^a^	ε (M^−1^cm^−1^)	λ_em_ (nm) ^b^	Τ (ns) ^c^	Φ ^d^	E_S1,exp_ (eV) ^e^	E_1/2_ (PC^•+^/PC) (V vs. SCE)	E^0^ (PC^•+^/^1^PC*) (V vs. SCE) ^e^	E^0^_calc_ (PC^•+^/^3^PC*) (V vs. SCE) ^f^
**PNH**	414	10,209	460	7.5	0.431	2.774	0.57	−2.20	−1.65
**PCH**	410	25,597	461	11.3	0.561	2.805	0.56	−2.25	−1.78
**POH**	435	13,405	470	10.8	0.505	2.805	0.74	−2.06	−1.51
**PSH**	412	13,111	474	6.0	0.368	2.774	0.77	−2.00	−1.43
**PSeH**	408	13,407	473	2.9	0.069	2.790	0.76	−2.03	−1.59/−2.04 ^g^

^a^ λmax (maximum absorption wavelength) measured in DCM. ^b,c^ Emission wavelength and lifetime were recorded by fluorescence spectroscopy in DCM. λ_ex_ = 450 nm. ^d^ Photoluminescence quantum efficiency (*Φ*) in solution was determined with excitation at corresponding λ_max_, and the error of the quantum yield measurement was in the range of 10% (three replicas). ^e^ Energy calculated at the lowest energy intersection (λ_int_) between normalized absorption and emission spectra, ***E_S1,exp_*** = 1240.5/λ_int_, *E*^0^ (PC^•+^/^1^PC*) = ***E*_1/2_** (**PC^•+^/PC**) − ***E_S1,exp_***. ^f^ Computed by DFT at uM06/6-311G(d)/SMD-DCM. ^g^ **PSeH** with uM06/LANL2DZ/SMD-DCM (see computational detail in the Supporting Information).

**Table 2:** Photoinduced polymerizations of MMA using different photocatalysts ^a^.

Entry	Photocatalyst	Conv. ^b^	*M*_n,theo_ (kg/mol) ^c^	*M*_n,GPC_ (kg/mol) ^d^	*Đ* ^d^	I* ^e^ (%)
1	**PNH**	64%	6.8	11.6	1.49	58
2	**POH**	80%	8.3	8.9	1.49	93
3	**PCH**	78%	8.1	12.3	1.27	66
4	**PSH**	67%	7.0	9.5	1.58	74
5 ^f^		45%	4.8	8.1	1.26	59
6 ^f^	**Nap-PTZ**	40%	4.2	7.0	1.31	60
7	**PSeH**	85%	8.7	8.0	1.24	111
8 ^g^		81%	8.4	9.8	1.32	85
9	**Nap-PSeZ**	85%	8.8	11.3	1.61	61

^a^ General conditions: MMA 9.35 mmol (1000 eq.), DBMM (initiator) 93.5 μmol (10 eq.), photocatalyst 9.35 μmol (0.1 eq.), PC loading at 100 ppm, DCM (same volume of monomer), irradiated with purple LEDs (385–395 nm) for 14–18 h. ^b^ Determined by ^1^H NMR. ^c^ *M*_n,theo_ = [monomer]/[initiator] × MW of monomer × Conv. % + MW of initiator. ^d^ *M*_n,GPC_ and *Ð* (*M*_w_*/M*_n_) were measured by GPC with PMMA standards, THF as eluent. ^e^ Initiator efficiency (I*) calculated by ((theoretical *M*_n_ by GPC-determined) × 100). ^f^ Irradiated with ultraviolet LEDs (365 nm) for 4 h. ^g^ Irradiated with white LEDs for 18 h.

**Table 3:** **PSeH**-mediated O-ATRP of different vinyl monomers ^a^.

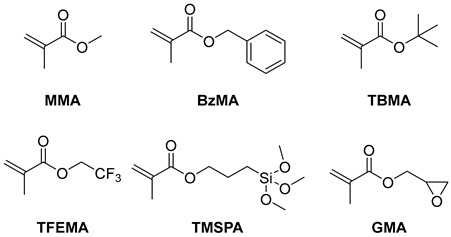						
Entry	Monomer	Solvent	Conv.	*M*_n,GPC_ (kg/mol)	*Đ*	I* (%)
1	BzMA	DMAc	84%	18.2	1.27	82
2	TFEMA	DCM	72%	14.8	1.33	84
3	TBMA	DCM	85%	17.8	1.51	70
4 ^b^	TMSPA	anisole	84%	12.0	1.39	80
5	GMA	DCM	57%	13.1	1.35	92
6	MMA	DCM	85%	8.0	1.24	111

^a^ Reaction conditions: [MMA]_0_:[DBMM]_0_:[**PSeH**]_0_ = 100:1:0.02, solvent (9.4 M of MMA), at room temperature, under the irradiation of purple LEDs for 7–48 h. Conv. values were determined by ^1^H NMR. *M*_n_ and *Đ* (*M*_w_*/M*_n_) were determined by GPC with PMMA standards. ^b^ [M]_0_/[DBMM]_0_ =50/1.

## Data Availability

The data presented in this study are available in article and Supplementary Materials.

## Supplementary Materials

The following supporting information can be downloaded at https://www.mdpi.com/article/10.3390/molecules29122763/s1. Synthesis of catalysts, NMR, UV-Vis, and emission spectra, computational detail, Figures S1–S19. Tables S1–S3. Figure S1. Reactors with 6W purple LEDs (up) and 6W white LEDs (down). Figure S2. ^1^H NMR of **PNH** in CDCl_3_. Figure S3. ^13^C NMR of **PNH** in CDCl_3_. Figure S4. ^1^H NMR of **PSH** in CDCl_3_. Figure S5. ^13^C NMR of **PSH** in CDCl_3_. Figure S6. ^1^H NMR of **POH** in CDCl_3_. Figure S7. ^13^C NMR of **POH** in CDCl_3_. Figure S8. ^1^H NMR of **PCH** in CDCl_3_. Figure S9. ^13^C NMR of **PCH** in CDCl_3_. Figure S10. ^1^H NMR of **PSeH** in CDCl_3_. Figure S11. ^13^C NMR of **PSeH** in CDCl_3_. Figure S12. UV spectra of catalysts **PNH**, **POH**, **PSH**, **PCH** and **PSeH** at different concentration in DCM. Figure S13. Figure S13. Fluorescence emission spectra of catalysts **PNH**, **POH**, **PSH**, **PCH** and **PSeH** in DCM. Figure S14. Fluorescence emission spectra of catalyst **PSeH** with DMAc, THF, benzotrifluoride, and toluene as solvent. Figure S15. Time-resolved emission decay curves of catalysts **PNH**, **PCH**, **POH**, **PSH** in DCM and **PSeH** in various solvent. Figure S16. Cyclic voltammograms (vs. Ag/AgCl) of catalysts **PNH**, **POH**, **PSH**, **PCH** and **PSeH** in MeCN. Figure S17. The SOMOs and redox potentials of triplet states for PC. Top figures show the low-lying singly occupied molecular orbital (SOMO) and bottom figures the higher-lying SOMO. Figure S18. Computed redox properties for PCs at uM06 / 6-311G(d) / SMD-DCM basis and uM06 / LANL2DZ / SMD-DCM basis. Figure S19. HOMO and LUMO energies of **PCs** and **Nap-PSeZ** calculated at the B3LYP/6-31G(d) level of theory. Table S1. Experimentally measured and theoretically computed excited state reduction potentials of **PCs**. Table S2. Sum of energies (0K) + ZPE, enthalpies and free energies of **PCs**. Table S3. HOMO & LUMO energies (in eV) and HOMO-LUMO gap (in eV) of the **PCs**.

## References

[1] Corrigan N., Jung K., Moad G., Hawker C.J., Matyjaszewski K., Boyer C. (2020). Reversible-Deactivation Radical Polymerization (Controlled/Living Radical Polymerization): From Discovery to Materials Design and Applications. Prog. Polym. Sci..

[2] Corrigan N., Yeow J., Judzewitsch P., Xu J., Boyer C. (2019). Seeing the Light: Advancing Materials Chemistry through Photopolymerization. Angew. Chem. Int. Ed..

[3] Walsh D.J., Hyatt M.G., Miller S.A., Guironnet D. (2019). Recent Trends in Catalytic Polymerizations. ACS Catal..

[4] de Ávila Gonçalves S., Rodrigues P.R., Pioli Vieira R. (2021). Metal-Free Organocatalyzed Atom Transfer Radical Polymerization: Synthesis, Applications, and Future Perspectives. Macromol. Rapid Commun..

[5] Huang Y.-S., Ejeta D.D., Kuo S.-W., Nakamura Y., Huang C.-F. (2023). Combinations (Є) among Controlled/Living Polymerizations and Utilizations of Efficient Chemical Reactions for the Synthesis of Novel Polymeric Materials. Polym. Chem..

[6] Wang J.-S., Matyjaszewski K. (1995). Controlled/”living” Radical Polymerization. Atom Transfer Radical Polymerization in the Presence of Transition-Metal Complexes. J. Am. Chem. Soc..

[7] Kato M., Kamigaito M., Sawamoto M., Higashimura T. (1995). Polymerization of Methyl Methacrylate with the Carbon Tetrachloride/Dichlorotris-(Triphenylphosphine)Ruthenium(II)/Methylaluminum Bis(2,6-di-*tert*-butylphenoxide) Initiating System: Possibility of Living Radical Polymerization. Macromolecules.

[8] Pan X., Tasdelen M.A., Laun J., Junkers T., Yagci Y., Matyjaszewski K. (2016). Photomediated Controlled Radical Polymerization. Prog. Polym. Sci..

[9] Corrigan N., Shanmugam S., Xu J., Boyer C. (2016). Photocatalysis in Organic and Polymer Synthesis. Chem. Soc. Rev..

[10] Dworakowska S., Lorandi F., Gorczyński A., Matyjaszewski K. (2022). Toward Green Atom Transfer Radical Polymerization: Current Status and Future Challenges. Adv. Sci..

[11] Treat N.J., Sprafke H., Kramer J.W., Clark P.G., Barton B.E., Read de Alaniz J., Fors B.P., Hawker C.J. (2014). Metal-Free Atom Transfer Radical Polymerization. J. Am. Chem. Soc..

[12] Pan X., Fang C., Fantin M., Malhotra N., So W.Y., Peteanu L.A., Isse A.A., Gennaro A., Liu P., Matyjaszewski K. (2016). Mechanism of Photoinduced Metal-Free Atom Transfer Radical Polymerization: Experimental and Computational Studies. J. Am. Chem. Soc..

[13] Theriot J., Lim C.-H., Yang H., Ryan M., Musgrave C., Miyake G. (2016). Organocatalyzed Atom Transfer Radical Polymerization Driven by Visible Light. Science.

[14] Pearson R.M., Lim C.-H., McCarthy B.G., Musgrave C.B., Miyake G.M. (2016). Organocatalyzed Atom Transfer Radical Polymerization Using N -Aryl Phenoxazines as Photoredox Catalysts. J. Am. Chem. Soc..

[15] McCarthy B., Miyake G.M. (2018). Organocatalyzed Atom Transfer Radical Polymerization Catalyzed by Core Modified N-Aryl Phenoxazines Performed under Air. ACS Macro Lett..

[16] Christensen J.A., Phelan B.T., Chaudhuri S., Acharya A., Batista V.S., Wasielewski M.R. (2018). Phenothiazine Radical Cation Excited States as Super-Oxidants for Energy-Demanding Reactions. J. Am. Chem. Soc..

[17] Liu Y., Chen Q., Tong Y., Ma Y. (2020). 9,9-Dimethyl Dihydroacridine-Based Organic Photocatalyst for Atom Transfer Radical Polymerization from Modifying “Unstable” Electron Donor. Macromolecules.

[18] Sartor S.M., Lattke Y.M., McCarthy B.G., Miyake G.M., Damrauer N.H. (2019). Effects of Naphthyl Connectivity on the Photophysics of Compact Organic Charge-Transfer Photoredox Catalysts. J. Phys. Chem. A.

[19] Buss B.L., Lim C.-H., Miyake G.M. (2020). Dimethyl Dihydroacridines as Photocatalysts in Organocatalyzed Atom Transfer Radical Polymerization of Acrylate Monomers. Angew. Chem. Int. Ed..

[20] Lattke Y.M., Corbin D.A., Sartor S.M., McCarthy B.G., Miyake G.M., Damrauer N.H. (2021). Interrogation of O-ATRP Activation Conducted by Singlet and Triplet Excited States of Phenoxazine Photocatalysts. J. Phys. Chem. A.

[21] Swisher N.A., Corbin D.A., Miyake G.M. (2021). Synthesis, Characterization, and Reactivity of N-Alkyl Phenoxazines in Organocatalyzed Atom Transfer Radical Polymerization. ACS Macro Lett..

[22] Corbin D.A., Cremer C., Puffer K.O., Newell B.S., Patureau F.W., Miyake G.M. (2022). Effects of the Chalcogenide Identity in N-Aryl Phenochalcogenazine Photoredox Catalysts. ChemCatChem.

[23] Wang G., Shao H., Ma J., Liao S. (2023). Chalcogenide-Doped Anthracenes as Organophotocatalysts for Metal-Free Atom Transfer Radical Polymerization. Macromol. Chem. Phys..

[24] Ma Q., Song J., Zhang X., Jiang Y., Ji L., Liao S. (2021). Metal-free Atom Transfer Radical Polymerization with ppm Catalyst Loading under Sunlight. Nat. Commun..

[25] Ma Q., Zhang X., Jiang Y., Lin J., Graff B., Hu S., Lalevée J., Liao S. (2022). Organocatalytic PET-RAFT Polymerization with a Low Ppm of Organic Photocatalyst under Visible Light. Polym. Chem..

[26] Zhang X., Jiang Y., Ma Q., Hu S., Liao S. (2021). Metal-Free Cation-ic Polymerization of Vinyl Ethers with Strict Temporal Control by Employing an Organophotocatalyst. J. Am. Chem. Soc..

[27] Dadashi-Silab S., Pan X., Matyjaszewski K. (2017). Phenyl Benzo[b]Phenothiazine as a Visible Light Photoredox Catalyst for Metal-Free Atom Transfer Radical Polymerization. Chem. A Eur. J..

[28] Discekici E.H., Anastasaki A., Read de Alaniz J., Hawker C.J. (2018). Evolution and Future Directions of Metal-Free Atom Transfer Radical Polymerization. Macromolecules.

[29] Peters A.T., Behesti Y.S.S. (1989). Benzo[*k, l*]xanthene-3, 4-dicarboximides and Benzimidazoxanthenoisoquinolinones—Yellow and Orange Dyes for Synthetic-polymer Fibres. J. Soc. Dye. Colour..

[30] Cremer C., Eltester M.A., Bourakhouadar H., Atodiresei I.L., Patureau F.W. (2021). Dehydrogenative C–H Phenochalcogenazination. Org. Lett..

[31] Zagranyarski Y., Skabeev A., Ma Y., Müllen K., Li C. (2016). Facile Synthesis of Annulated Heterocyclic Benzo[Kl]Acridine Derivatives via One-Pot N–H/C–H Coupling. Org. Chem. Front..

[32] Singh V.K., Yu C., Badgujar S., Kim Y., Kwon Y., Kim D., Lee J., Akhter T., Thangavel G., Park L.S. (2018). Highly Efficient Organic Photocatalysts Discovered via a Computer-Aided-Design Strategy for Visible-Light-Driven Atom Transfer Radical Polymerization. Nat. Catal..

[33] Wu C., Corrigan N., Lim C.-H., Liu W., Miyake G., Boyer C. (2022). Rational Design of Photocatalysts for Controlled Polymerization: Effect of Structures on Photocatalytic Activities. Chem. Rev..

[34] Winget P., Cramer C.J., Truhlar D.G. (2004). Computation of Equilibrium Oxidation and Reduction Potentials for Reversible and Dissociative Electron-Transfer Reactions in Solution. Theor. Chem. Acc..

[35] Zhao Y., Truhlar D.G. (2008). The M06 Suite of Density Functionals for Main Group Thermochemistry, Thermochemical Kinetics, Noncovalent Interactions, Excited States, and Transition Elements: Two New Functionals and Systematic Testing of Four M06-Class Functionals and 12 Other Functionals. Theor. Chem. Acc..

[36] He H., Zapol P., Curtiss L.A. (2010). A Theoretical Study of CO_2_ Anions on Anatase (101) Surface. J. Phys. Chem. C.

[37] Roth H., Romero N., Nicewicz D. (2015). Experimental and Calculated Electrochemical Potentials of Common Organic Molecules for Applications to Single-Electron Redox Chemistry. Synlett.

[38] Lee K., Serdiuk I.E., Kwon G., Min D.J., Kang K., Park S.Y., Kwon J.E. (2020). Phenoxazine as a High-Voltage p-Type Redox Center for Organic Battery Cathode Materials: Small Structural Reorganization for Faster Charging and Narrow Operating Voltage. Energy Environ. Sci..

[39] Weiss R., VanOrman Z.A., Sullivan C.M., Nienhaus L. (2022). A Sensitizer of Purpose: Generating Triplet Excitons with Semiconductor Nanocrystals. ACS Mater. Au.

[40] Malinge A., Kumar S., Chen D., Zysman-Colman E., Kéna-Cohen S. (2024). Heavy Atom Effect in Halogenated mCP and Its Influence on the Efficiency of the Thermally Activated Delayed Fluorescence of Dopant Molecules. J. Phys. Chem. C.

