# Supplementary material for: The Development of Visible-Light Organic Photocatalysts for Atom Transfer Radical Polymerization via Conjugation Extension
Source: Molecules. 2024 Jun 11;29(12):2763. doi: 10.3390/molecules29122763 (PMC11206499; doi:10.3390/molecules29122763)
Supplement: Supplementary file 1 [file molecules-29-02763-s001.zip › molecules-3026011-supplementary.pdf]

Supplementary Information

The Development of Visible Light Organic Photocatalysts for Atom Transfer Radical Polymerization via Conjugation Extension

Hui Shao[1], Runzhi Long[1], Hui Xu[1], Pan Sun[1], Guangrong Wang[1], Yuanming Li[1]*, and Saihu Liao[1,2]*

[1]Key Laboratory of Molecule Synthesis and Function Discovery (Fujian Province University), College of Chemistry, Fuzhou University, Fuzhou 350108, China

[2]State Key Laboratory of Physical Chemistry of Solid Surfaces, College of Chemistry and Chemical Engineering, Xiamen University, Xiamen 361005, China

*Author to whom correspondence should be addressed: yuanming.li@fzu.edu.cn (Y.L.); shliao@xmu.edu.cn (S.L.)

Light source

All polymerization reactions were conducted in a 6 W blue photo-reactor placed 1 cm from light, which was purchased from http://www.geaochem.com/ (Model: H106062, GEAO CHEMICAL). The reactor has a fan for cooling and its light intensity to be ~30 mW/cm^2.

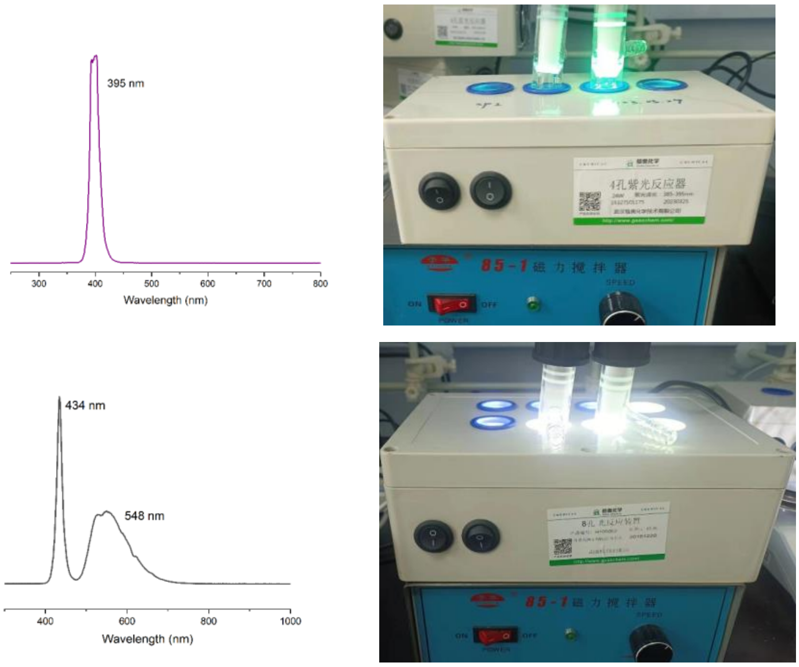

Figure S1. Reactors with 6W Purple LEDs(up) and 6W white LEDs(down).

Synthesis and Characterization of Photocatalysts

A stirred mixture of a 2-bromoaniline (1.13 ml, 10 mmol, 1 eq.), 2-bromoiodobenzene (1.54 ml, 12 mmol, 1.2 eq.), NaO*t*-Bu (1.34 g, 14 mmol, 1.4 eq.), Pd2(dba)3 (0.46 g, 0.5 mmol, 5 mol%) and DPPF (5.54 g, 1 mmol, 10 mol%) in toluene were heated to 120°C for 18 h. Until 2-bromoaniline was totally consumed by moternitting by TLC.

The solvent was removed in vacuo. And the reaction mixture was extracted with ethyl acetate and dried over Na_2SO_4. The crude product was purified by flash column chromatography. Then, a screw-cap vial is charged with bis(2-bromophenyl)amine(1.64 g, 5 mmol, 1 eq.), CuI (0.1 g, 0.5 mmol, 10 mol%) and NaI (3.03 g, 20 mmol, 4 eq.), evacuated and backfilled with Argon. N,N'-Dimethylethylendiamine (108 μL, 1 mmol, 20 mol%) and dry dioxane were added under inert atmosphere. The reaction mixture was stirred for 24 h at 110°C. The reaction mixture was extracted with DCM, the organic layer was dried over Na_2SO_4 and concentrated in vacuo. The crude product was purified by flash column chromatography.

Bis(2-iodophenyl)amine, Se powder (2 eq.), KOH (4 eq.) were dissolved in dry DMSO under Argon atmosphere. The reaction mixture was stirred at 120°C for 24 h. After reaching room temperature, the reaction mixture was diluted with sat. NH_4Cl solution and DCM. The aqueous layer was extracted with DCM. The combined organic layer was washed with sat. NH_4Cl and brine and dried over Na_2SO_4. The solvent was removed on silica. The crude product was purified by flash column chromatography using hexane/dichloromethane (6:4) yielding the compound **RM 5** as a pale brown solid.

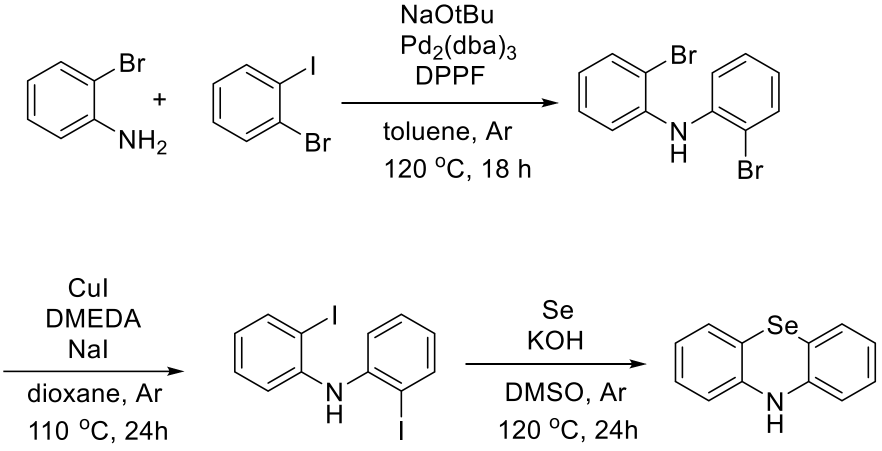

RM 5

10H-phenoselenazine **RM5** were prepared as described in the reference [*Org. Chem. Front.* **2016**, *3*, 1520].

General Procedure for PXH: A mixture of dibromoaryl compound (3.5 mmol), 10H-phenochalcogenazines (3 mmol), sodium *tert*-butoxyde (7 mmol, 0.67 g), $Pd(OAc)_2$ (3 mol%, 0.09 mmol, 0.02 g), $Pd_2(dba)_3$ (3 mol%, 0.09 mmol, 0.082 g), tricyclohexylphosphine (7 mol%, 0.21 mmol, 0.06 g) and tri-*tert*-butylphosphine (7 mol%, 0.21 mmol, 0.043 g) in 50 ml dry toluene was stirred at 90 °C for 10 h. The solvent was removed under reduced pressure and the residue was purified by column chromatography using hexane/dichloromethane as eluent on silica.

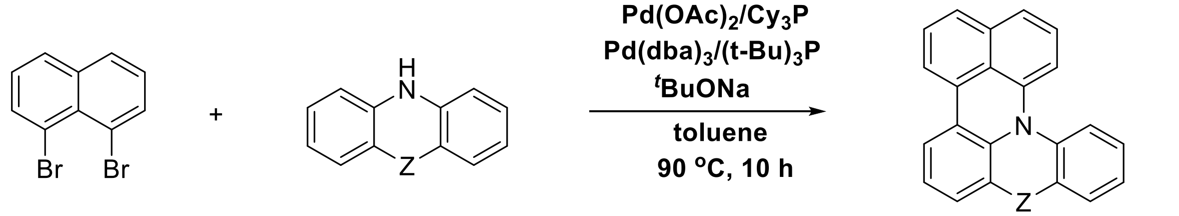

RM 1 Z = H
RM 2 Z = $C(CH_3)_2$
RM 3 Z = S
RM 4 Z = O
RM 5 Z = Se

PNH Z = H
PCH Z = $C(CH_3)_2$
PSH Z = S
POH Z = O
PSeH Z = Se

7-phenyl-7H-benzo[kl]acridine

Yellow solid. Yield from diphenylamine 0.69 g (78%).

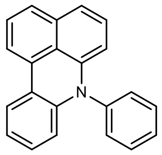

PNH:

^{1}**H NMR** (500 MHz, $CDCl_3$) δ 7.91 (dd, J = 8.0, 1.5 Hz, 1H), 7.70 (d, J = 7.8 Hz, 2H), 7.67 – 7.59 (m, 1H), 7.61 – 7.52 (m, 1H), 7.46 (dd, J = 8.3, 1.1 Hz, 1H), 7.39 (t, J = 7.8 Hz, 1H), 7.39 – 7.31 (m, 2H), 7.10 – 6.97 (m, 3H), 6.93 (td, J = 7.6, 7.1, 1.2 Hz, 1H), 6.22 (dd, J = 8.3, 1.2 Hz, 1H), 5.77 (dd, J = 7.4, 1.4 Hz, 1H).

^{13}**C NMR** (126 MHz, $CDCl_3$) δ 142.1, 141.7, 140.4, 135.9, 131.7, 130.8, 130.7, 129.2, 128.8, 127.4, 127.2, 124.6, 124.2, 123.1, 121.1, 121.0, 116.9, 115.4, 113.4, 104.9.

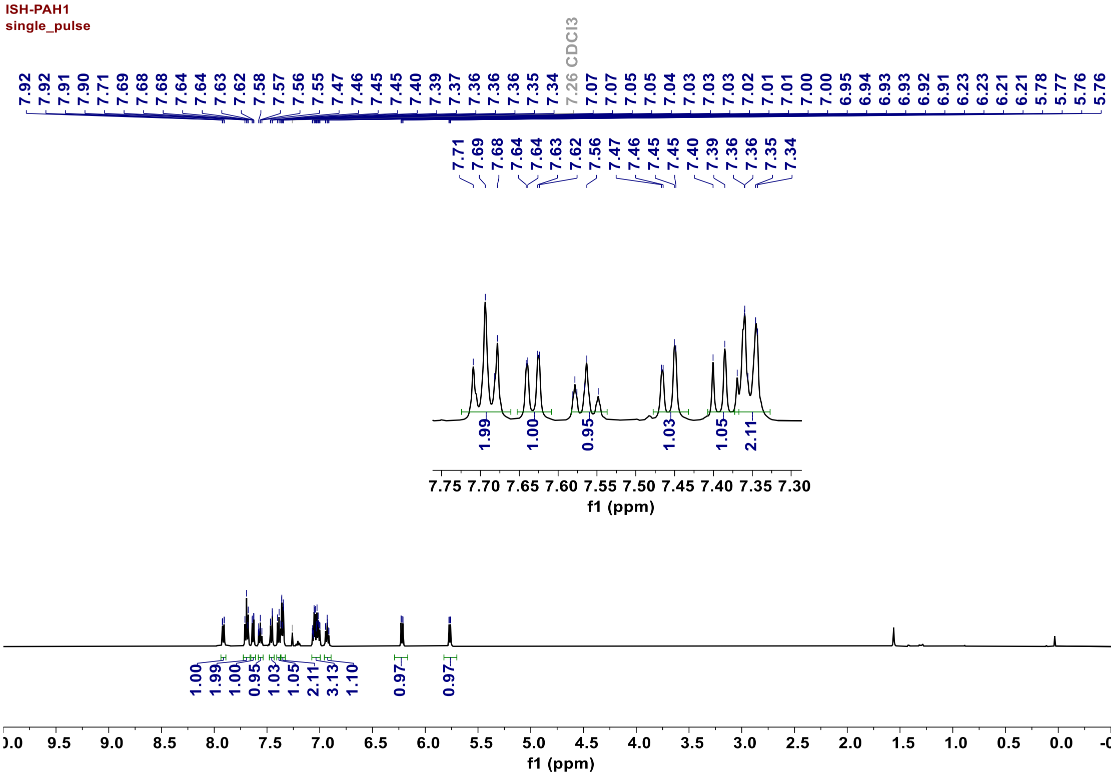

Figure S2. ^{1}H NMR of **PNH** in $CDCl_3$.

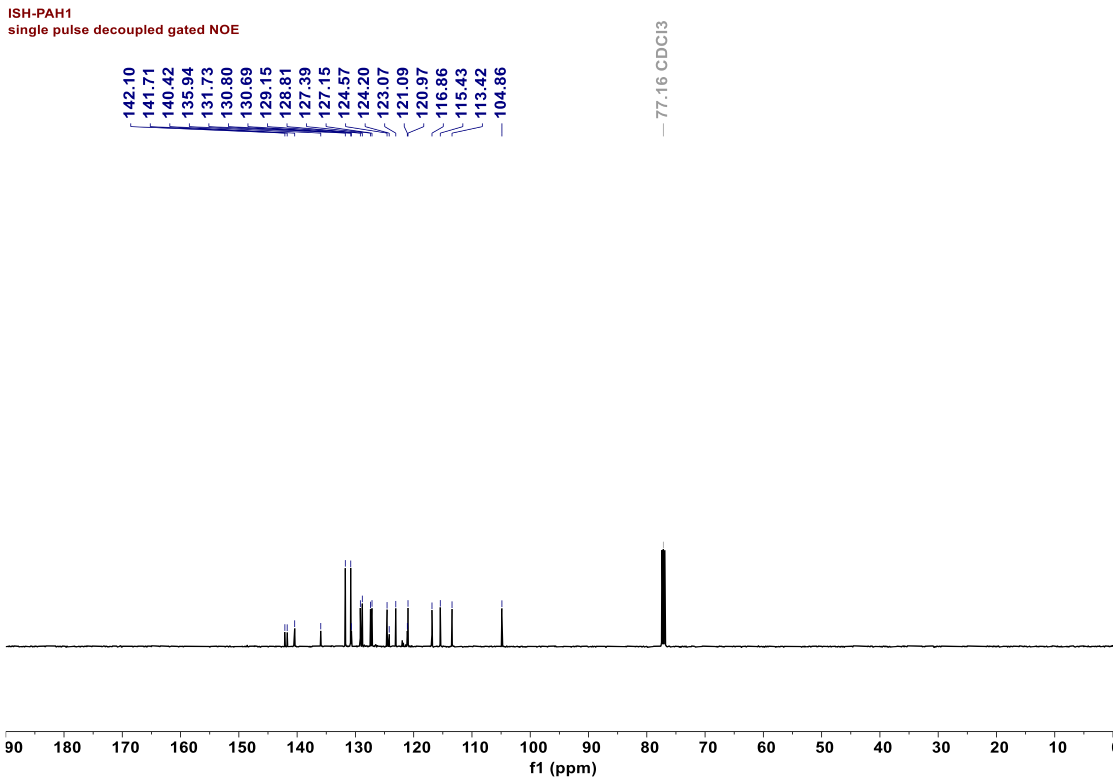

Figure S3. ^{13}C NMR of **PNH** in $CDCl_3$.

benzo[4,5]quinolino[3,2,1-kl]phenothiazine

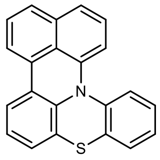

PSH:

Yellow crystal solid. Yield from 10H-phenothiazine 0.68 g (70%).

^{1}H NMR (500 MHz, $CDCl_3$) δ 7.70 (d, J = 6.9 Hz, 1H), 7.67 (dd, J = 7.9, 1.3 Hz, 1H), 7.61 (d, J = 8.3 Hz, 1H), 7.53 (dd, J = 8.0, 1.6 Hz, 1H), 7.44 (dd, J = 8.2, 7.4 Hz, 1H), 7.39 (dd, J = 7.1, 2.1 Hz, 1H), 7.34 – 7.30 (m, 3H), 7.16 (dd, J = 7.6, 1.4 Hz, 1H), 7.12 – 7.02 (m, 3H).

^{13}C NMR (126 MHz, $CDCl_3$) δ 144.1, 140.7, 137.9, 135.3, 129.0, 128.9, 127.7, 127.5, 127.3, 127.2, 127.0, 126.5, 126.2, 125.1, 124.6, 124.5, 122.1, 120.9, 118.3, 116.1, 113.6.

HRMS (m/z): calcd for $[M+H]^+$ $C_{22}H_{14}NS$, 324.0841; found 324.0836.

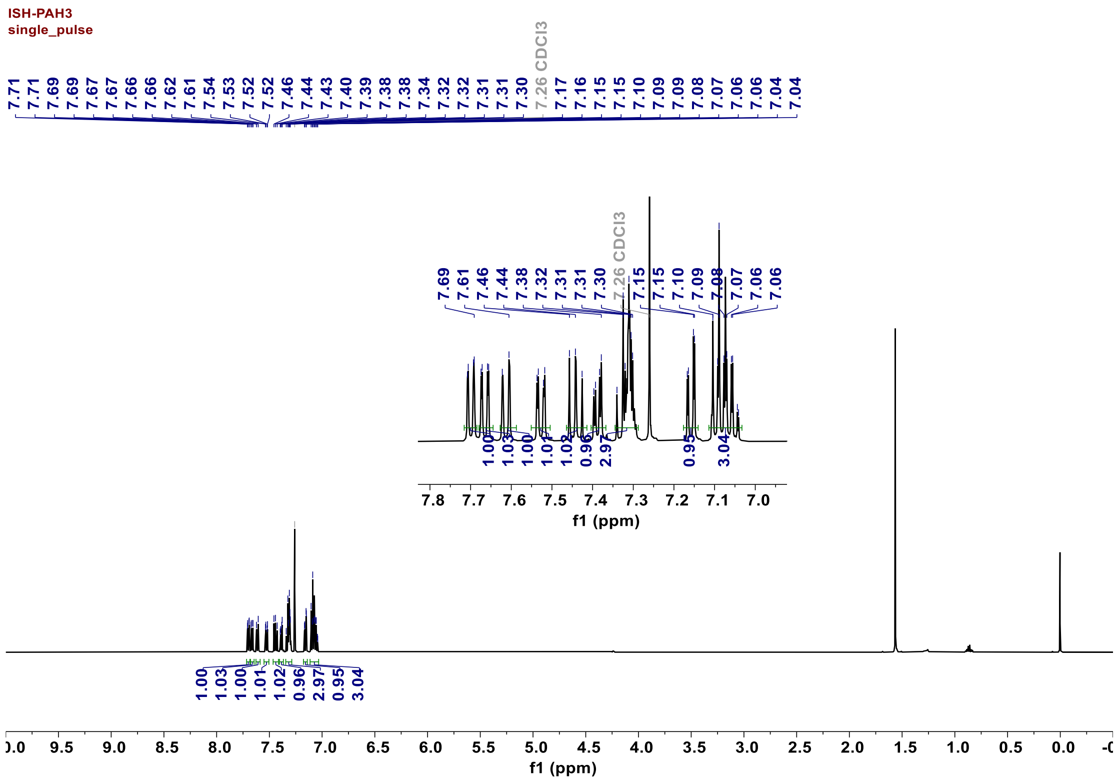

Figure S4. ^{1}H NMR of **PSH** in $CDCl_3$.

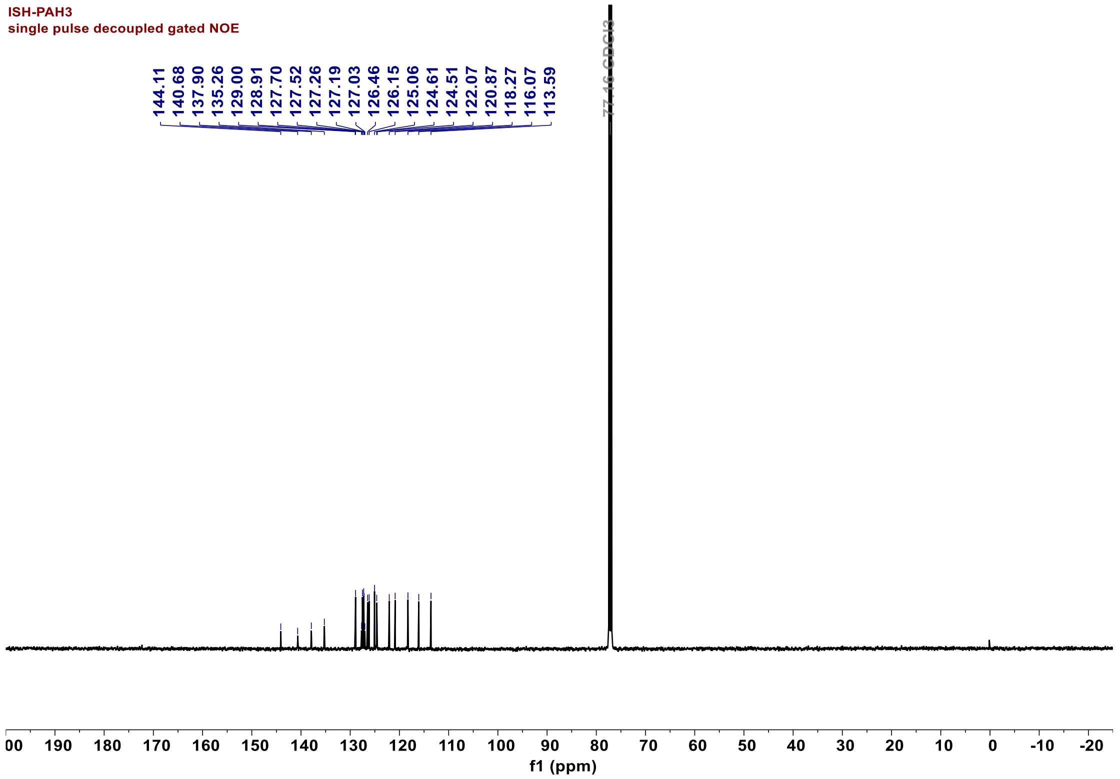

Figure S5. ^{13}C NMR of **PSH** in $CDCl_3$.

benzo[4,5]quinolino[3,2,1-kl]phenoxazine

Yellow crystal solid. Yield from 10H-phenoxazine 0.41 g (45%).

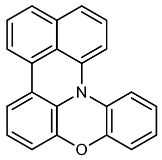

POH:

[1]**H NMR** (500 MHz, $CDCl_3$) δ 7.80 (dd, *J* = 8.1, 1.2 Hz, 1H), 7.59 (d, *J* = 7.1 Hz, 1H), 7.55 (d, *J* = 7.6 Hz, 1H), 7.44 – 7.38 (m, 3H), 7.30 (d, *J* = 7.2 Hz, 2H), 7.03 – 6.97 (m, 3H), 6.96 – 6.92 (m, 1H), 6.84 (dd, *J* = 7.9, 1.2 Hz, 1H).

[13]**C NMR (126 MHz, $CDCl_3$)** δ 148.7, 146.8, 135.7, 135.2, 131.4, 130.3, 128.8, 128.0, 127.2, 126.3, 125.8, 124.7, 123.9, 123.5, 120.4, 119.7, 118.1, 117.8, 115.4, 114.3, 109.2.

HRMS (m/z): calcd for $[M+H]^+$ $C_{22}H_{14}NO$, 308.1069; found 308.1063.

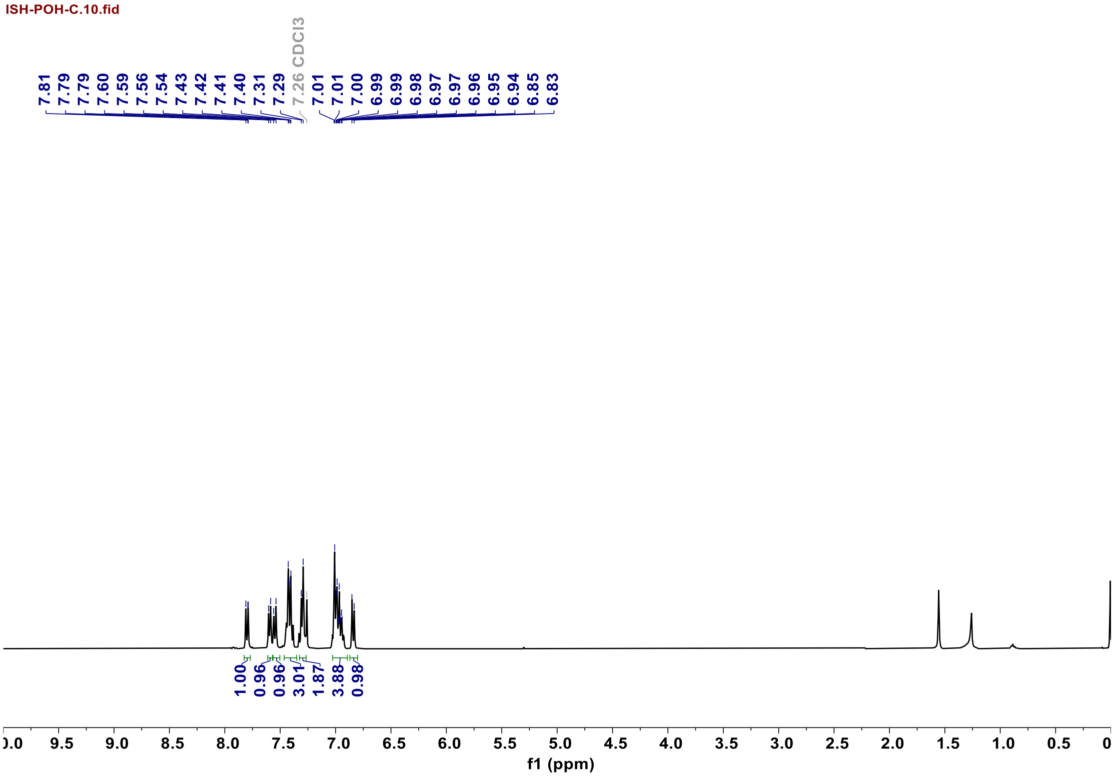

Figure S6. ^{1}H NMR of **POH** in $CDCl_3$.

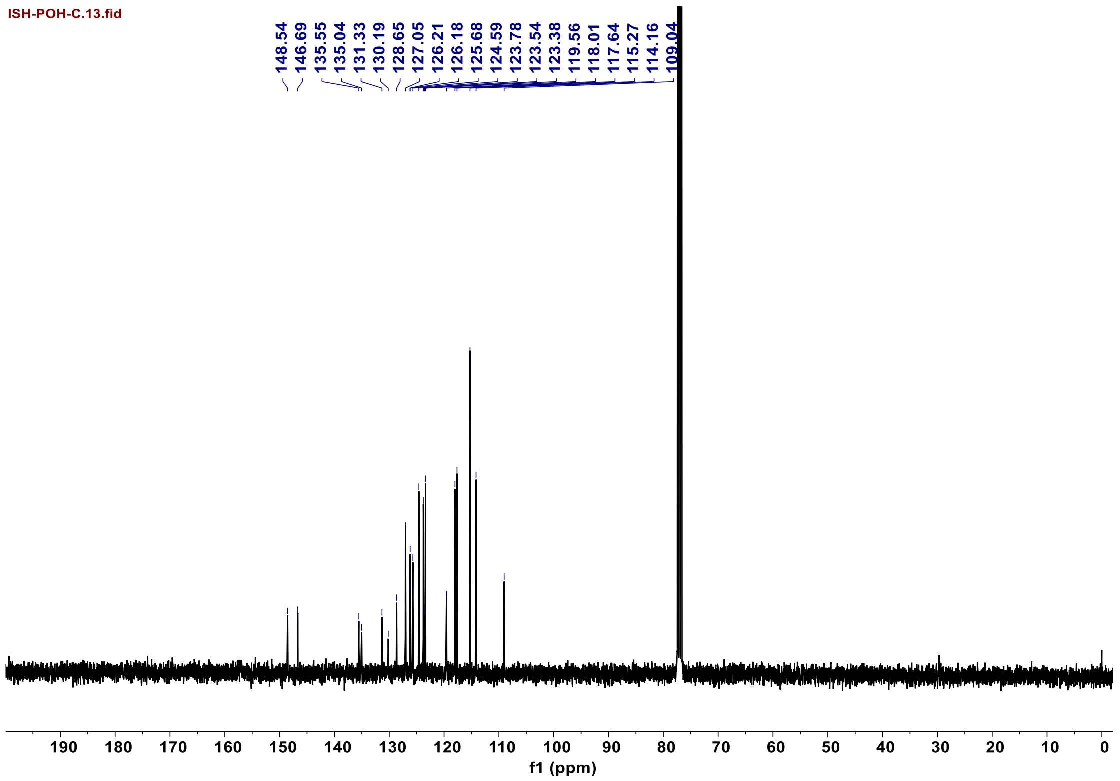

Figure S7. ^{13}C NMR of **POH** in $CDCl_3$.

7,7-dimethyl-7H-benzo[kl]quinolino[3,2,1-de]acridine

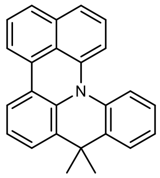

PCH:

Yellow crystal solid. Yield from 9,9-dimethyl-9,10-dihydroacridine 0.58 g (58%).
^{1}H NMR (400 MHz, $CDCl_3$) δ 7.95 – 7.93 (m, 1H), 7.72 (ddd, J = 11.6, 7.7, 1.2 Hz, 2H), 7.58 (dd, J = 8.2, 1.0 Hz, 1H), 7.52 – 7.47 (m, 1H), 7.43 (t, J = 8.2, 7.3 Hz, 1H), 7.41 – 7.40 (m, 1H), 7.34 – 7.32 (m, 3H), 7.16 – 7.13 (m, 3H), 1.92 (s, 3H), 1.36 (s, 3H).

^{13}C NMR (101 MHz, $CDCl_3$) δ 140.2, 137.3, 137.1, 136.9, 135.5, 134.8, 129.8, 127.1, 126.7, 126.3, 126.1, 125.3, 124.1, 124.0, 123.3, 123.3, 123.2, 120.7, 119.7, 115.5, 115.3, 111.5, 36.8, 30.4, 23.1.

HRMS (m/z): calcd for $[M+H]^+$ $C_{25}H_{20}N$, 334.1594; found 334.1590.

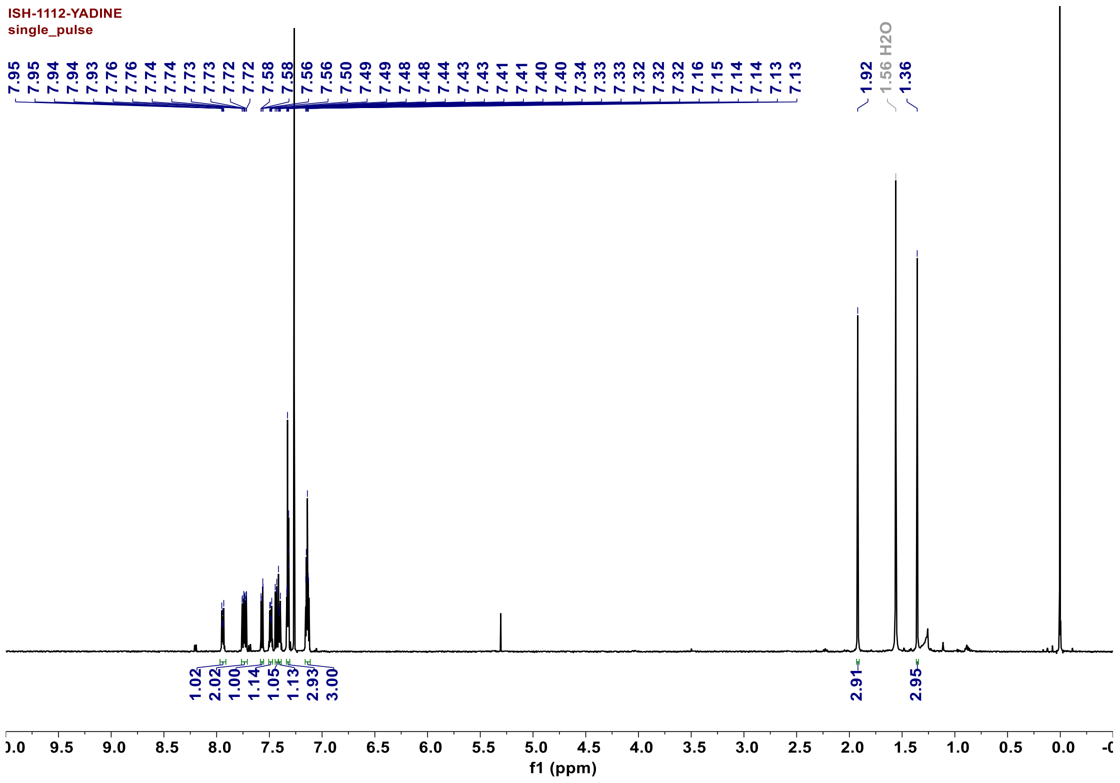

Figure S8. ^{1}H NMR of **PCH** in $CDCl_3$.

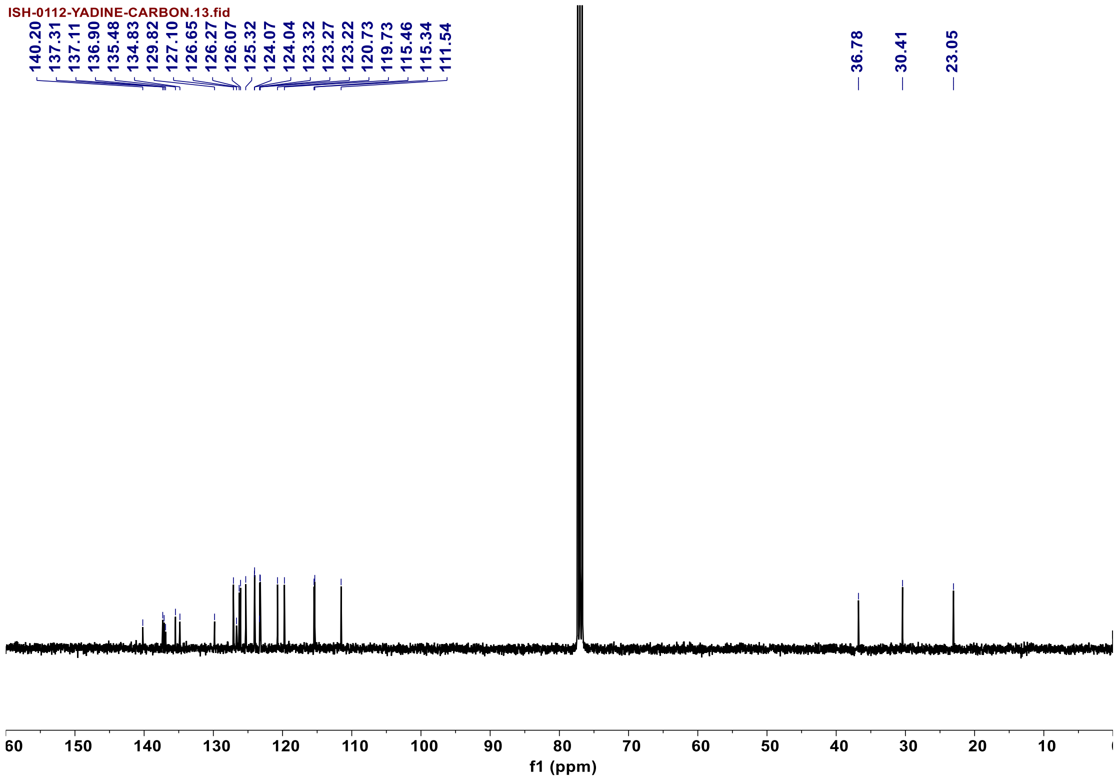

Figure S9. ^{13}C NMR of **PCH** in $CDCl_3$.

benzo[4,5]quinolino[3,2,1-kl]phenoselenazine

Yellow crystal solid. Yield from 10H-phenoselenazine 0.79 g (71%).

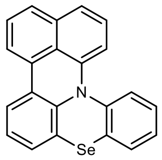

PSeH:

^{1}**H NMR** (500 MHz, $CDCl_3$) δ 7.73 (t, J = 7.6 Hz, 2H), 7.63 (d, J = 8.2 Hz, 1H), 7.49 – 7.44 (m, 1H), 7.40 (d, J = 8.2 Hz, 2H), 7.35 – 7.30 (m, 2H), 7.21 (d, J = 7.6 Hz, 1H), 7.13 – 7.07 (m, 2H), 7.05 (td, J = 7.5, 1.0 Hz, 2H).

^{13}**C NMR** (126 MHz, $CDCl_3$) δ 144.4, 140.4, 138.1, 134.2, 131.3, 129.8, 129.2, 127.9, 127.2, 126.6, 126.2, 125.9, 125.3, 125.0, 124.3, 122.5, 121.5, 120.7, 120.4, 116.2, 114.1**.**

HRMS (m/z): calcd for $[M+H]^+$ $C_{22}H_{14}NSe$, 372.0286; found 372.0271.

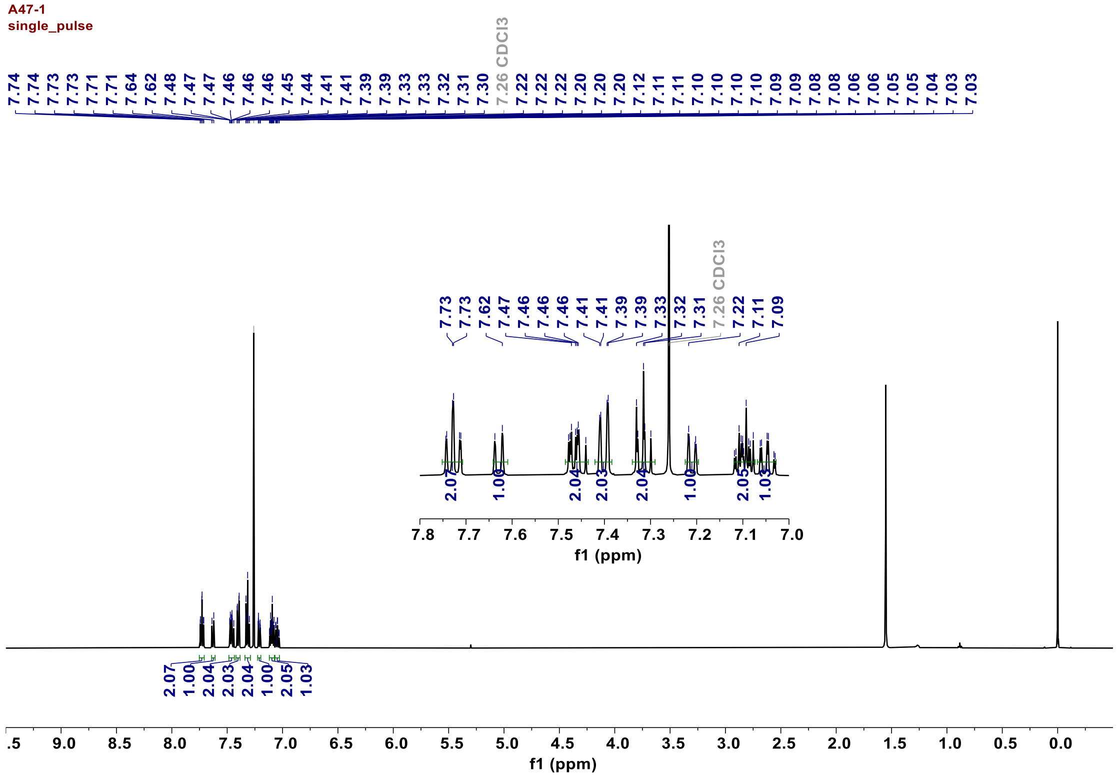

Figure S10. ^{1}H NMR of **PSeH** in $CDCl_3$.

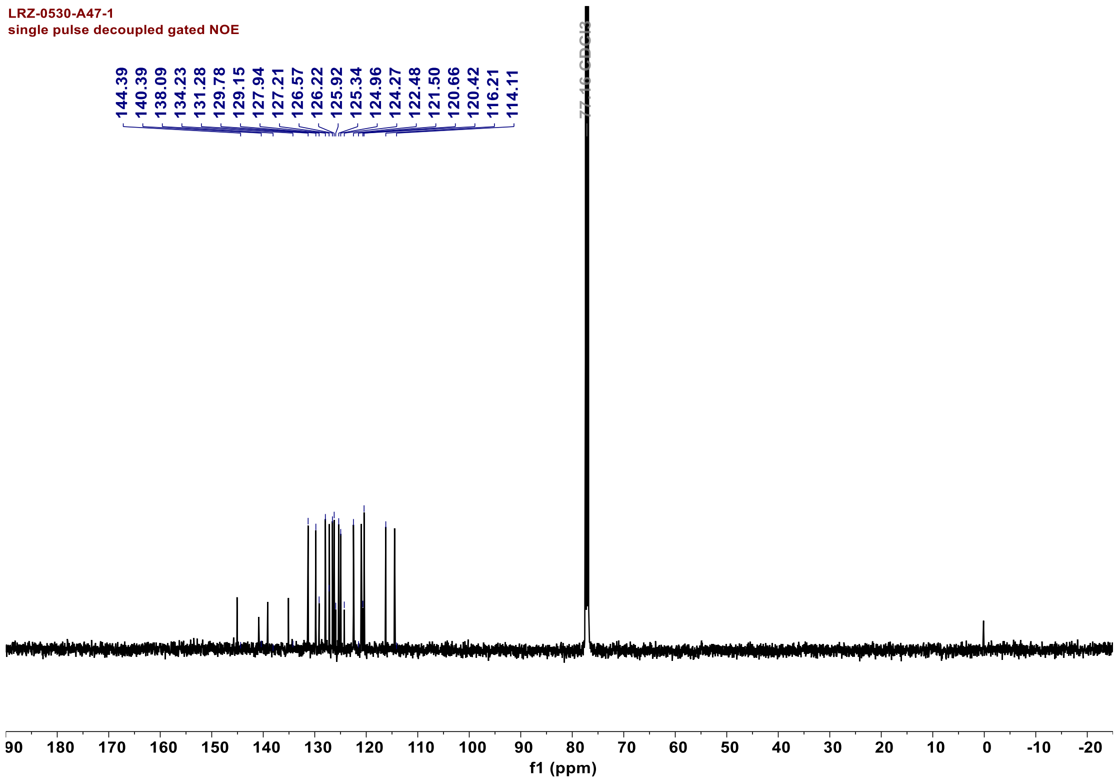

Figure S11. ^{13}C NMR of **PSeH** in $CDCl_3$.

UV-Vis Absorption Spectra

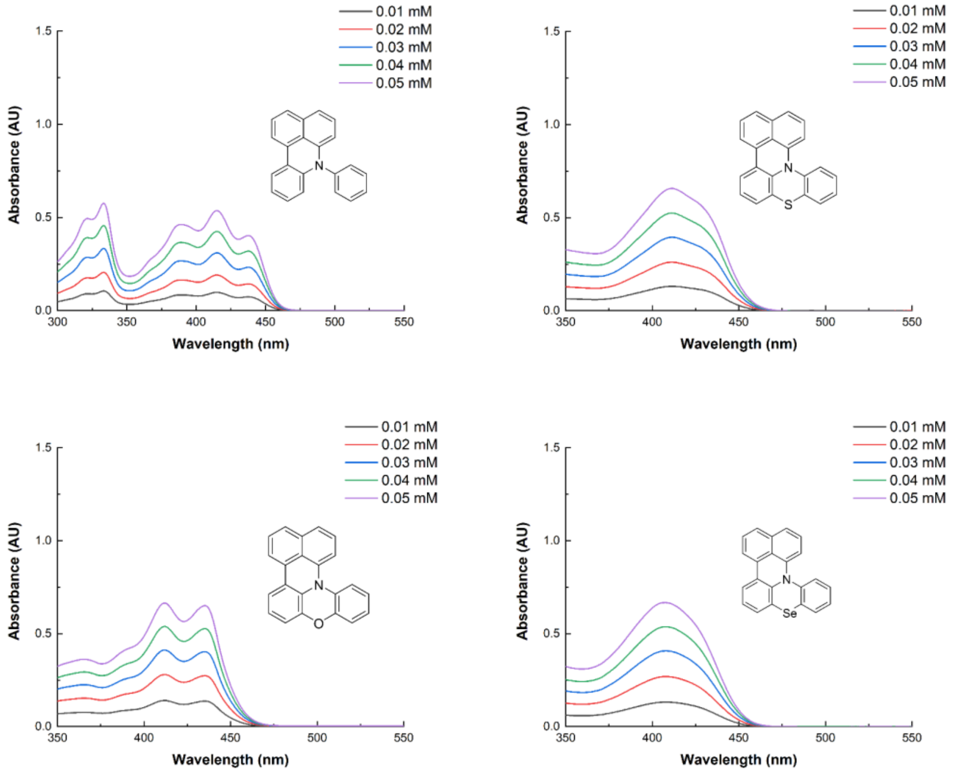

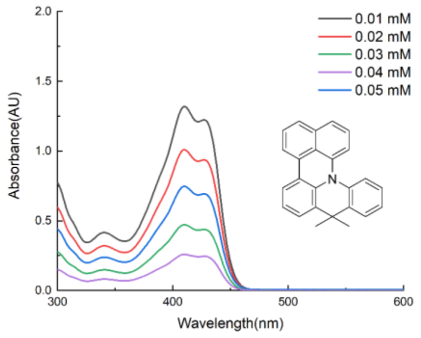

Figure S12. UV spectra of catalysts **PNH**, **POH**, **PSH**, **PCH** and **PSeH** at different concentration in DCM.

Fluorescence Emission Spectra

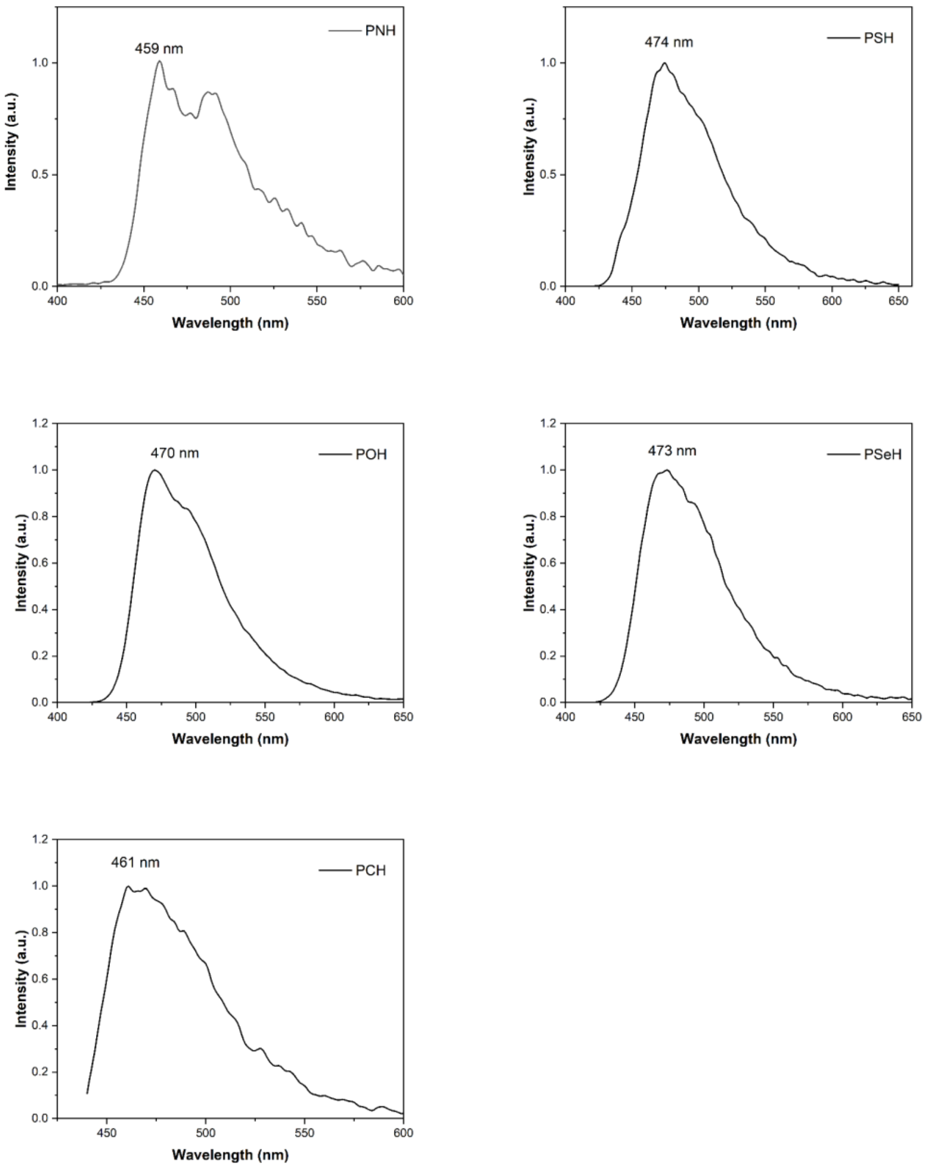

Figure S13. Fluorescence emission spectra of catalysts **PNH**, **POH**, **PSH**, **PCH** and **PSeH** in DCM.

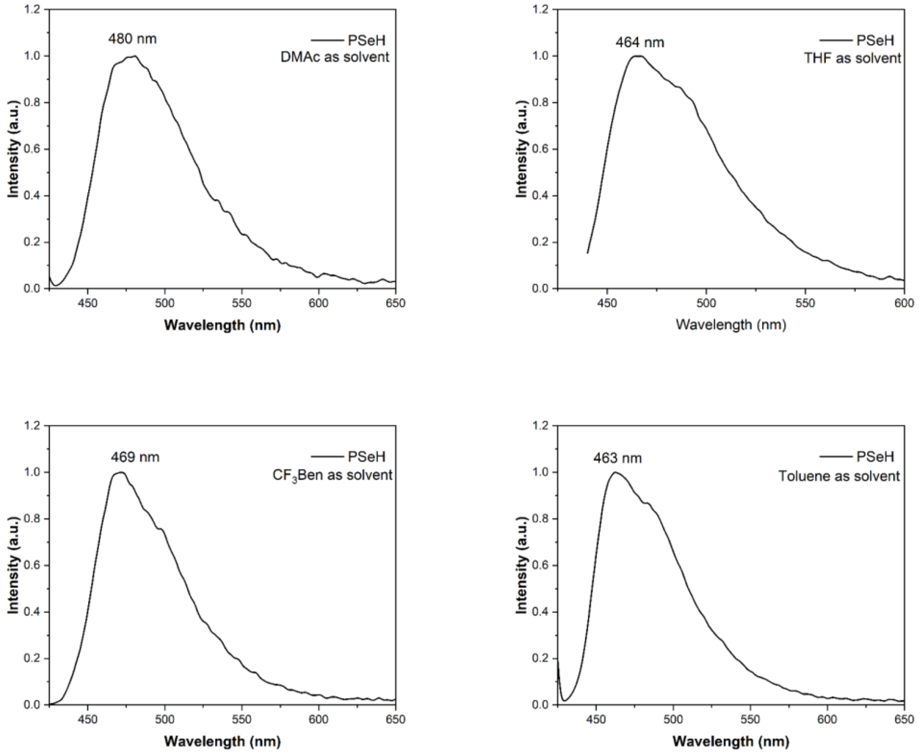

Figure S14. Fluorescence emission spectra of catalyst **PSeH** with DMAc, THF, benzotrifluoride, and toluene as solvent.

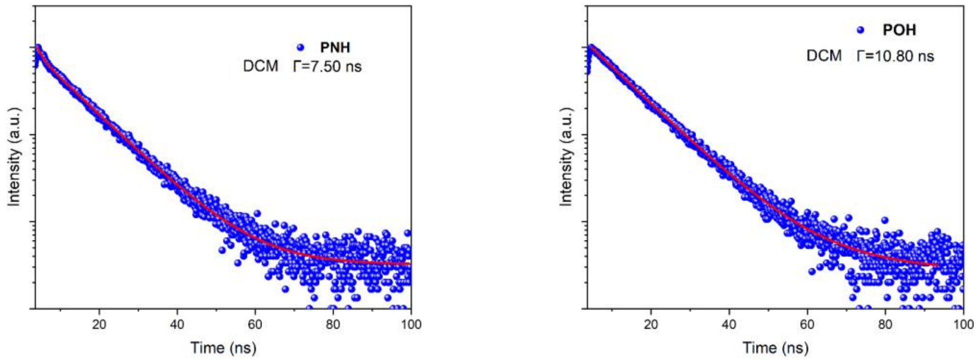

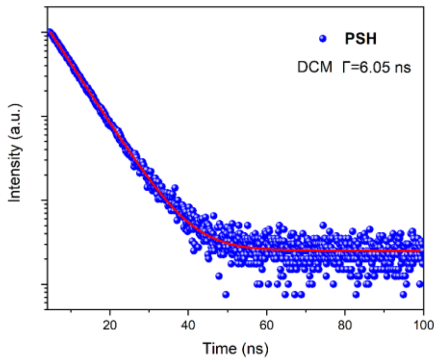
PSH
DCM Γ=6.05 ns
Intensity (a.u.)
20
40
60
80
100
Time (ns)

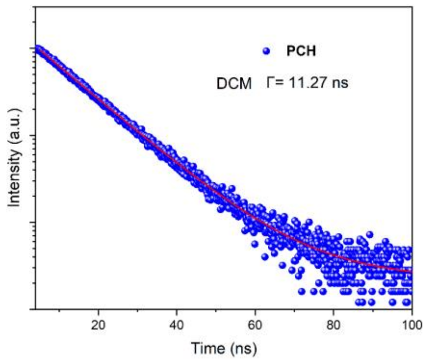
PCH
DCM Γ= 11.27 ns
Intensity (a.u.)
20
40
60
80
100
Time (ns)

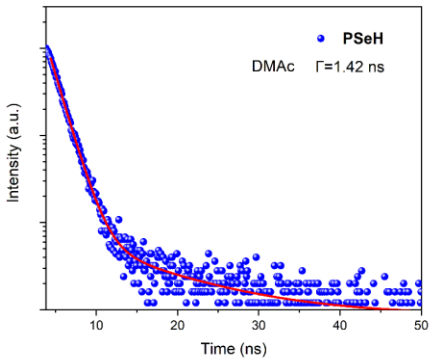
PSeH
DMAc Γ=1.42 ns
Intensity (a.u.)
10
20
30
40
50
Time (ns)

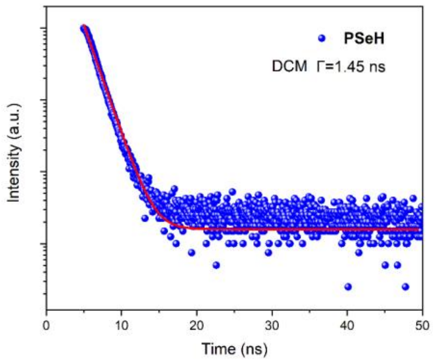
PSeH
DCM Γ=1.45 ns
Intensity (a.u.)
0
10
20
30
40
50
Time (ns)

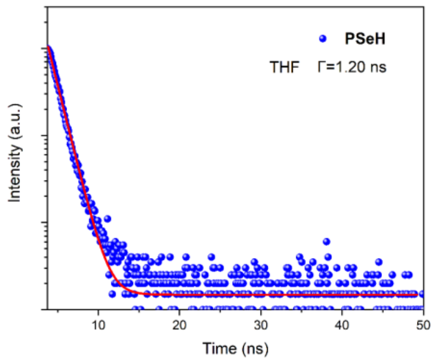
PSeH
THF Γ=1.20 ns
Intensity (a.u.)
10
20
30
40
50
Time (ns)

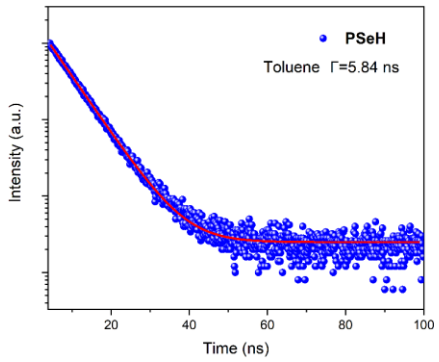
PSeH
Toluene Γ=5.84 ns
Intensity (a.u.)
20
40
60
80
100
Time (ns)

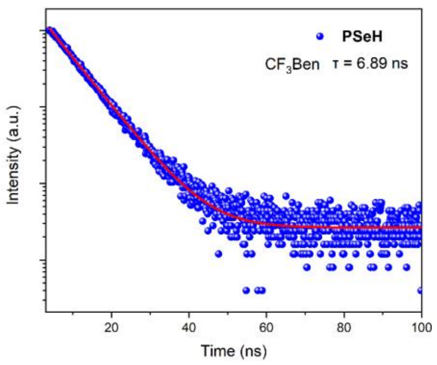
PSeH
CF3Ben τ = 6.89 ns
Intensity (a.u.)
20
40
60
80
100
Time (ns)

Figure S15. Time-resolved emission decay curves of catalysts **PNH**, **PCH**, **POH**, **PSH** in DCM and **PSeH** in different solvent.

Cyclic Voltammetry

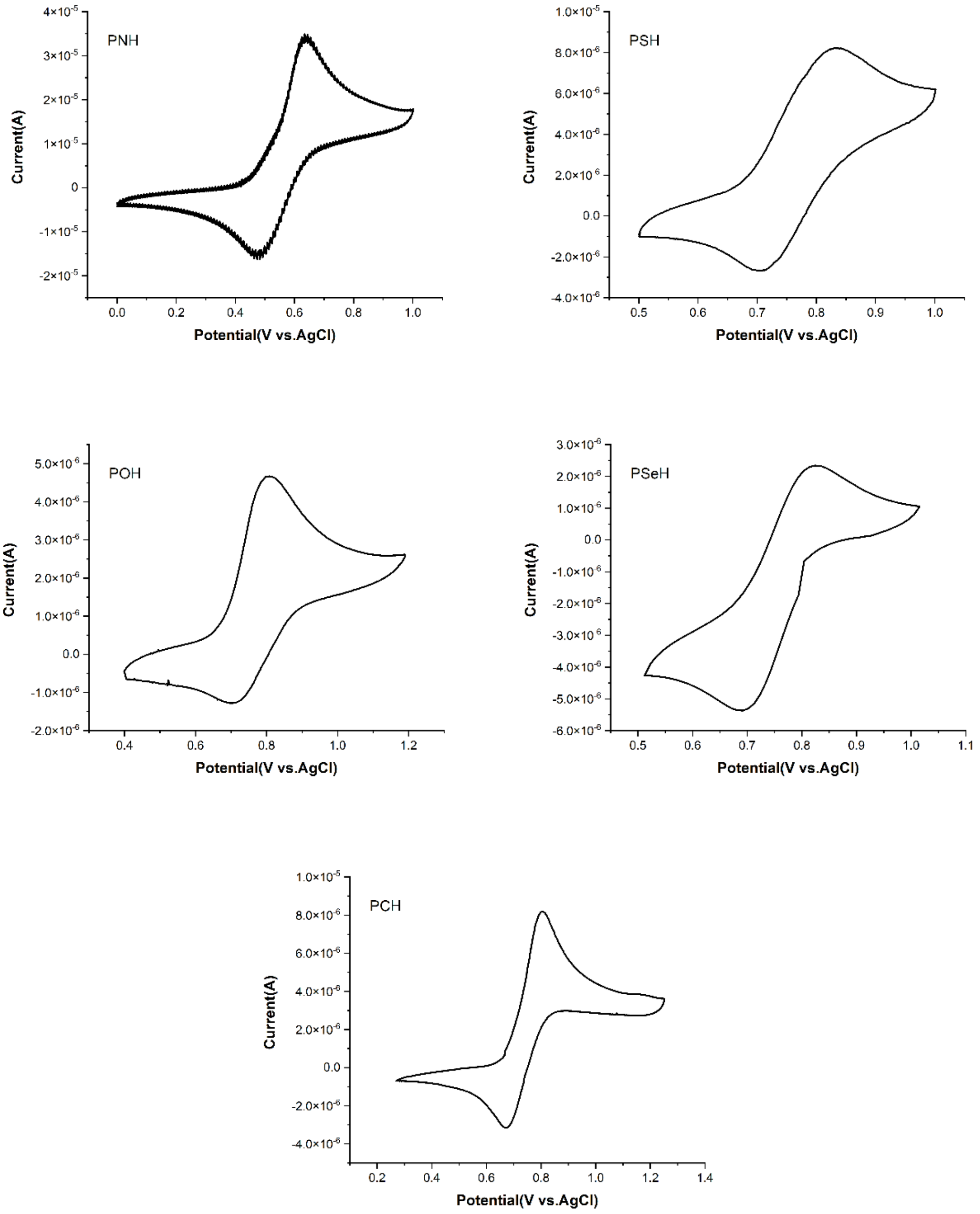

Figure S16. Cyclic voltammograms (vs. Ag/AgCl) of catalysts **PNH**, **POH**, **PSH**, **PCH** and **PSeH** in MeCN.

Computational Detail

Singly occupied molecular orbitals (SOMOs) of the triplet excited state ($^3PC^*$) were generated from the optimized structures utilizing Gaussian 16 at M06/6-31+G** in SMD model (for **PNH**, **PCH**, **POH**, and **PSH**) and at M06/Lanl2dz in SMD model (for **PSeH** and **Nap PSeZ**). However, due to the highly expensive computational cost to calculate Gibbs free energies ($G^{\Theta}_{(solvent)}$) of all the compounds at B3LYP/6-311+G* level, $G^{\Theta}_{(solvent)}$ values of $^3PC^*$, $PC^{\bullet+}$ and PC were combined with thermochemical corrections calculated *via* frequency analyses at B3LYP/6-31G* level in gas phase without scaling to produce improved estimates of the solvent phase ΔG^{Θ} listed in the text and schemes ($G^{\Theta}_{(solvent)} = G^{\Theta}_{(gas)} + (E^{\Theta}_{(solvent)} - E^{\Theta}_{(gas)})$). The single point electronic energies ($E^{\Theta}_{(solvent)}$) of $^3PC^*$, $PC^{\bullet+}$ and PC were calculated at the M06/6-311+G* level of theory in dichloromethane using geometries optimized at B3LYP/6-31G* level of theory in gas phase. The frontier orbital energies are calculated by B3LYP/6-31G(d) level of theory with Gaussian 16 program. Visualization of the results was performed by the use of GaussView 5.0.9 software.

Reduction potentials calculation

Standard reduction potentials (E^0) were calculated by the following previously reported procedures [1, 2, 10]. A value of -100.5 kcal/mol was assumed for the reduction free energy of the standard hydrogen electrode (SHE). Thus, $E^0 = (-100.5 - \Delta G_{red})/23.06$ (V vs. SHE); for E^0 ($PC^{\bullet+}$ / $^3PC^*$), $\Delta G_{red} = G(^3PC^*) - G(PC^{\bullet+})$ while for E^0 ($PC^{\bullet+}$ / PC), $\Delta G_{red} = G(PC) - G(PC^{\bullet+})$. To reference to the Saturated Calomel Electrode (SCE), E^0 (vs. SHE) is converted to E^0 (vs. SCE) using E^0 (vs. SCE) = E^0 (vs. SHE) - 0.24 V. Triplet energies (in eV) of PCs were obtained by [$G(^3PC^*) - G(PC)$, in kcal/mol]/23.06.

Coordinates of molecular structures

All coordinates are reported as XYZ Cartesian coordinates. The single point electronic energies of $^3PC^*$, $PC^{\bullet+}$, and PC (for **PNH**, **PCH**, **POH**, and **PSH**) were calculated at the b3lyp/6-311+G* level of theory in SMD-DCM solvent using geometries optimized at b3lyp /6-31G* level of theory in gas phase. The enthalpies and Gibbs free energies of $^3PC^*$, $PC^{\bullet+}$, and PC (for **PNH**, **PCH**, **POH**, and **PSH**) were calculated at the b3lyp/6-31G* level of theory in gas phase. Enthalpies and Gibbs free energies in solution phase were obtained as described in the Computation details section. Energies are reported in parentheses with the following order: E^{Θ}, H^{Θ}, G^{Θ}. They are stated in Hartree unit. All energies reported were calculated using the density functional theory (DFT) calculations using Gaussian16.

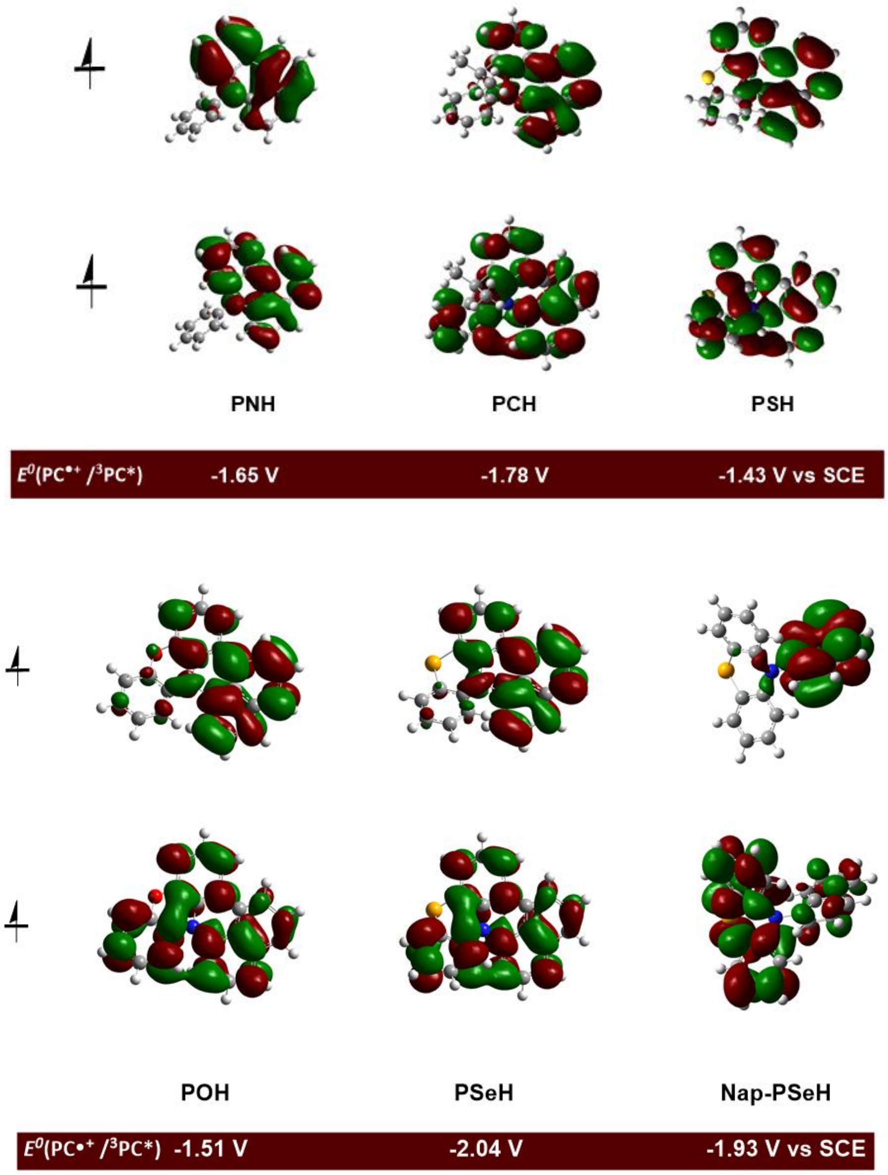

Figure S17. The SOMOs and redox potentials of triplet states for PC. Top figures show the low-lying singly occupied molecular orbital (SOMO) and bottom figures the higher-lying SOMO.

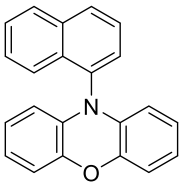

uM06/LANL2DZ
E_{T1} = 1.99 eV
E^0 ($PC^{\bullet+}/^1PC^*$) = -2.14 V vs. SCE
E^0 ($PC^{\bullet+}/^1PC$) = -0.15 V vs. SCE

uM06/6-311G(d)
E_{T1} = 2.42 eV
E^0 ($PC^{\bullet+}/^1PC^*$) = -1.97 V vs. SCE
E^0 ($PC^{\bullet+}/^1PC$) = 0.45 V vs. SCE

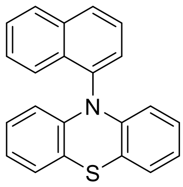

uM06/LANL2DZ
E_{T1} = 2.03 eV
E^0 ($PC^{\bullet+}/^1PC^*$) = -2.08 V vs. SCE
E^0 ($PC^{\bullet+}/^1PC$) = -0.04 V vs. SCE

uM06/6-311G(d)
E_{T1} = 2.60 eV
E^0 ($PC^{\bullet+}/^1PC^*$) = -2.14 V vs. SCE
E^0 ($PC^{\bullet+}/^1PC$) = 0.46 V vs. SCE

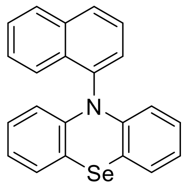

uM06/LANL2DZ
E_{T1} = 2.38 eV
E^0 ($PC^{\bullet+}/^1PC^*$) = -1.93 V vs. SCE
E^0 ($PC^{\bullet+}/^1PC$) = 0.45 V vs. SCE

uM06/6-311G(d)
E_{T1} = 2.65 eV
E^0 ($PC^{\bullet+}/^1PC^*$) = -2.32 V vs. SCE
E^0 ($PC^{\bullet+}/^1PC$) = 0.33 V vs. SCE

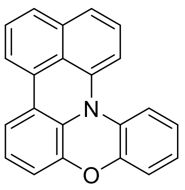

uM06/LANL2DZ
E_{T1} = 1.46 eV
E^0 ($PC^{\bullet+}/^1PC^*$) = -1.69V vs. SCE
E^0 ($PC^{\bullet+}/^1PC$) = -0.23V vs. SCE

uM06/6-311G(d)
E_{T1} = 1.88 eV
E^0 ($PC^{\bullet+}/^1PC^*$) = -1.51 V vs. SCE
E^0 ($PC^{\bullet+}/^1PC$) = 0.36 V vs. SCE

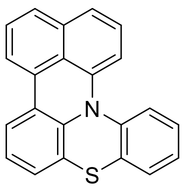

uM06/LANL2DZ
E_{T1} = 1.43 eV
E^0 ($PC^{\bullet+}/^1PC^*$) = -1.58 V vs. SCE
E^0 ($PC^{\bullet+}/^1PC$) = -0.14 V vs. SCE

uM06/6-311G(d)
E_{T1} = 1.87 eV
E^0 ($PC^{\bullet+}/^1PC^*$) = -1.43 V vs. SCE
E^0 ($PC^{\bullet+}/^1PC$) = 0.34 V vs. SCE

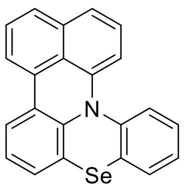

uM06/LANL2DZ
E_{T1} = 2.46 eV
E^0 ($PC^{\bullet+}/^1PC^*$) = -2.04 V vs. SCE
E^0 ($PC^{\bullet+}/^1PC$) = 0.42 V vs. SCE

uM06/6-311G(d)
E_{T1} = 2.02 eV
E^0 ($PC^{\bullet+}/^1PC^*$) = -1.59 V vs. SCE
E^0 ($PC^{\bullet+}/^1PC$) = 0.43 V vs. SCE

Figure S18. Computed redox properties for PCs at uM06 / 6-311G(d) / SMD-DCM basis and uM06 / LANL2DZ / SMD-DCM basis.

Table S1. Experimentally measured and theoretically computed excited state reduction potentials of **PCs.**

PC	E(triplet), theo (V vs. SCE)a),c)	$E_{1/2}$ ($PC^{\bullet+}/PC$) (V vs. SCE)b)	E^0 ($PC^{\bullet+}/PC$), theo (V vs. SCE)a),d)	E^{0*} ($PC^{\bullet+}/^1PC^*$) (V vs. SCE)	E^{0*} ($PC^{\bullet+}/^3PC^*$), theo (V vs. SCE)a),e)
**PNH**	1.99	0.57	0.35	-2.20	-1.65
**PCH**	1.89	0.56	0.11	-2.25	-1.78
**POH**	1.88	0.74	0.42	-2.06	-1.51
**PSH**	1.87	0.77	0.44	-2.00	-1.43
**PSeH**	2.46	0.76	0.42	-2.03	-2.04

[a]. Theoretical predictions from DFT calculations at the M06/6-311+G* level in SMD-DCM model (see Computational details section). [b] Obtained from cyclic voltammetry. [c] Calculated triplet energy. [d] Calculated oxidation potential of $^{2}PC^{\bullet+}$. [e] Calculated triplet state reduction potential $^{3}PC^{*}$.

Table S2. Sum of energies (0K) + ZPE, enthalpies and free energies of **PCs.**

**PC**	**Nap-PXZ**	**PNH**	**PCH**	**POH**	**PSH**	**PSeH**	**Nap-PSeZ**
**E**	-976.52908	-901.34499	-1017.95785	-975.37968	-1298.35899	-909.36093	-910.51866
**H**	-976.52814	-901.34404	-1017.95691	-975.37874	-1298.35804	-909.35998	-910.51772
**G**	-976.59308	-901.40832	-1018.02138	-975.43777	-1298.41862	-909.42110	-910.58422

**$^{3}PC^{*}$**	**Nap-PXZ**	**PNH**	**PCH**	**POH**	**PSH**	**PSeH**	**Nap-PSeZ**
**E**	-976.43949	-901.27205	-1017.88687	-975.30898	-1298.28837	-909.26870	-910.42777
**H**	-976.43855	-901.27111	-1017.88593	-975.30804	-1298.28742	-909.26776	-910.42683
**G**	-976.50431	-901.33508	-1017.95203	-975.36871	-1298.34981	-909.33164	-910.49661

**$PC^{\bullet+}$**	**Nap-PXZ**	**PNH**	**PCH**	**POH**	**PSH**	**PSeH**	**Nap-PSeZ**
**E**	-976.34472	-901.16597	-1017.78449	-975.19685	-1298.17335	-909.17600	-910.33292
**H**	-976.34378	-901.16503	-1017.78354	-975.19590	-1298.17241	-909.17506	-910.33197
**G**	-976.40766	-901.22663	-1017.84833	-975.25533	-1298.23356	-909.23747	-910.39850

Table S3. HOMO & LUMO energies (in eV) and HOMO-LUMO gap (in eV) of the **PCs**.

Compound	Energies(eV)		
	LUMO	HOMO	E(LUMO)-E(HOMO)
**PNH**	-1.3761	-4.8431	3.47
**PCH**	-1.7091	-4.9976	3.42
**POH**	-1.6226	-4.9971	3.38
**PSH**	-1.6819	-5.1296	3.45
**PSeH**	-1.5685	-5.0831	3.52
**Nap-PTZ**	-1.6760	-5.2347	3.56
**Nap-PSeZ**	-1.4308	-5.3171	3.89

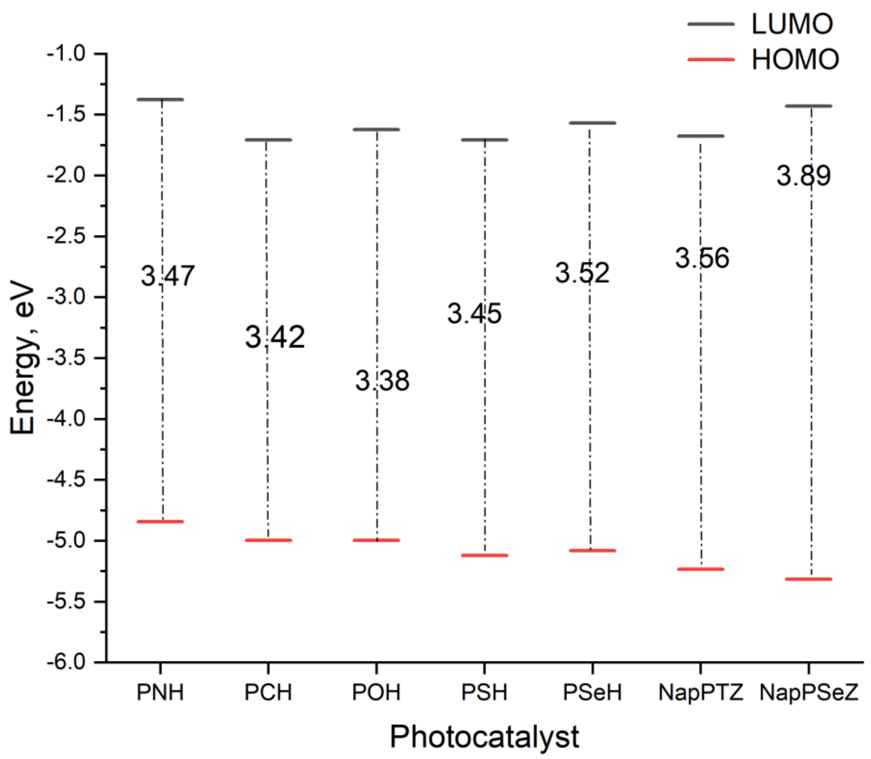

Figure S19. HOMO and LUMO energies of **PCs** and **Nap-PSeZ** calculated at the B3LYP/6-31G(d) level of theory.

Computational Geometries and Energies

PNH

Singlet state

C	4.39107400	-0.10820400	-0.00263200
C	3.44981100	0.92928300	-0.00267000
C	2.09202000	0.66240200	-0.00085300
C	1.65995100	-0.69320900	0.00028600
C	2.61806500	-1.74825100	0.00050800
C	3.99118200	-1.41804700	-0.00086300
C	0.27386400	-1.02267500	0.00091400
C	-0.11719800	-2.35143900	0.00212100
C	0.84230800	-3.37377900	0.00269000
C	2.18124100	-3.09231600	0.00186300
C	1.07037200	1.71180100	0.00006500
N	-0.66247000	0.00929600	-0.00005200
C	1.39287400	3.07269600	0.00115200
C	0.42358200	4.05712300	0.00168400
C	-0.91660500	3.68859200	0.00120800
C	-1.27264600	2.35378400	0.00054400
C	-0.29240400	1.35253800	0.00015400
C	-2.05354700	-0.33311900	-0.00050900
C	-2.71798900	-0.50230900	-1.20717300
C	-4.06386100	-0.84220400	-1.20552500
C	-4.73641800	-1.01192000	-0.00152500
C	-4.06501900	-0.84127800	1.20300100
C	-2.71915300	-0.50140200	1.20566600
H	5.45095900	0.13782600	-0.00402100
H	3.81052100	1.95367700	-0.00428600
H	4.72172100	-2.22540100	-0.00067200
H	-1.17068700	-2.61310800	0.00251700
H	0.50191000	-4.40728200	0.00372400
H	2.92469000	-3.88716800	0.00211100
H	2.43712200	3.37319600	0.00180300
H	0.71017400	5.10560600	0.00254800
H	-1.69639700	4.44681900	0.00155400
H	-2.32389500	2.08206600	0.00039100
H	-2.17215900	-0.36615900	-2.13910100
H	-4.58940200	-0.97586200	-2.14811200
H	-5.79067500	-1.27895300	-0.00195000
H	-4.59142200	-0.97420400	2.14520700
H	-2.17418900	-0.36453600	2.13799500

Energies (0K) = -901.361358

Energies (0K) + ZPE = -901.344987
Enthalpies (298K) = -901.344042
Free Energies (298K) = -901.408317

Cation radical

C	4.38938700	-0.11008100	-0.02962500
C	3.46482100	0.92030100	-0.02892800
C	2.08391300	0.65777500	-0.00732800
C	1.65388600	-0.68665100	0.00336300
C	2.60387400	-1.74472200	0.00497700
C	3.97304800	-1.42991100	-0.01018600
C	0.27223900	-1.00683500	0.01077800
C	-0.13559800	-2.35643400	0.02433800
C	0.80517400	-3.36964800	0.03017000
C	2.15957300	-3.08143200	0.02011500
C	1.08830200	1.69731900	0.00298500
N	-0.64868800	0.00437300	0.00281700
C	1.41311200	3.06545300	0.01591800
C	0.43805500	4.03065200	0.01796300
C	-0.91334000	3.66139900	0.00697000
C	-1.27487300	2.33858600	-0.00010100
C	-0.28470800	1.33951800	0.00142000
C	-2.05582600	-0.33394900	-0.00350300
C	-2.70025000	-0.50133900	-1.21835500
C	-4.04877800	-0.82878000	-1.21959400
C	-4.72883200	-0.98188500	-0.01778300
C	-4.06641000	-0.80873700	1.19117100
C	-2.71792100	-0.48127200	1.20442200
H	5.45022500	0.12353500	-0.04568900
H	3.82513600	1.94357900	-0.04843900
H	4.69823400	-2.24073700	-0.00883400
H	-1.19138800	-2.60512900	0.03093800
H	0.46946100	-4.40241200	0.04209000
H	2.89684800	-3.88093900	0.02350100
H	2.45417400	3.37127800	0.02813000
H	0.71437800	5.08114600	0.02831700
H	-1.68587500	4.42554200	0.00626900
H	-2.32439600	2.06538600	-0.00556800
H	-2.14646600	-0.37486400	-2.14625700
H	-4.56918500	-0.96529500	-2.16392300
H	-5.78515000	-1.23870700	-0.02338600
H	-4.60059500	-0.92969300	2.12990100
H	-2.17767600	-0.33926300	2.13810000

Energies (0K) = -901.182126

Energies (0K) + ZPE = -901.165972
Enthalpies (298K) = -901.165027
Free Energies (298K) = -901.226631

triplet state

C	4.40176600	-0.11161100	0.00009700
C	3.50434900	0.91178600	0.00006900
C	2.07202700	0.65831000	-0.00005600
C	1.63823900	-0.71110100	-0.00000700
C	2.59033700	-1.77115000	-0.00002300
C	3.97517900	-1.46005000	0.00000000
C	0.27560500	-1.02378000	-0.00000500
C	-0.15373400	-2.37570500	-0.00009400
C	0.77394600	-3.38952000	-0.00012700
C	2.13985300	-3.09601500	-0.00008000
C	1.10973100	1.68478500	-0.00012000
N	-0.65683200	0.00474900	0.00004600
C	1.43481200	3.07031100	-0.00036400
C	0.46079900	4.04448000	-0.00007300
C	-0.88914000	3.69857600	0.00042800
C	-1.25380700	2.35369800	0.00048600
C	-0.28715700	1.35387700	0.00013000
C	-2.05444900	-0.32246500	-0.00000400
C	-2.71901500	-0.47808900	-1.20765400
C	-4.06843800	-0.80350600	-1.20458800
C	-4.74163800	-0.96679200	-0.00011000
C	-4.06833400	-0.80434800	1.20442500
C	-2.71891500	-0.47892400	1.20759700
H	5.46756200	0.10802300	0.00021800
H	3.86746300	1.93520400	0.00028400
H	4.69824900	-2.27284900	0.00003400
H	-1.21323600	-2.61118100	-0.00015200
H	0.43973900	-4.42371600	-0.00020000
H	2.87316300	-3.90083200	-0.00010200
H	2.47839500	3.37060000	-0.00085900
H	0.75309300	5.09277000	-0.00024300
H	-1.65924500	4.46548500	0.00074800
H	-2.30662900	2.08781200	0.00077000
H	-2.17181900	-0.34563300	-2.13912900
H	-4.59554200	-0.93192900	-2.14688900
H	-5.79848500	-1.22307400	-0.00015500
H	-4.59535700	-0.93342300	2.14668400
H	-2.17162800	-0.34712600	2.13911300

Energies (0K) = -901.288866

Energies (0K) + ZPE = -901.272053
Enthalpies (298K) = -901.271109
Free Energies (298K) = -901.335085

POH

Singlet state

C	-4.34978200	0.59624000	0.72832500
C	-3.21158200	1.41675000	0.69557500
C	-1.98885100	0.91724800	0.29329600
C	-1.87944300	-0.46280800	-0.04091300
C	-3.04541600	-1.27926700	-0.06536300
C	-4.27801600	-0.71413700	0.33564600
C	-0.62351600	-1.04917600	-0.36904100
C	-0.57213400	-2.35048800	-0.82757100
C	-1.73725900	-3.12965600	-0.88589500
C	-2.94676400	-2.62358100	-0.48911000
C	-0.80383900	1.75648000	0.10765600
N	0.52920200	-0.26798000	-0.21127400
C	-0.84031800	3.15636400	0.11282300
C	0.30021500	3.90179300	-0.11872600
C	1.51307400	3.26908800	-0.37732000
C	1.55490200	1.89189200	-0.40802100
C	0.41431400	1.12787800	-0.16305500
C	1.81071500	-0.78818600	0.09043600
C	2.91309500	0.02254900	-0.18588800
C	4.20345400	-0.39456000	0.08067700
C	4.41757700	-1.64287400	0.64871000
C	3.33014000	-2.44020900	0.97819600
C	2.03600600	-2.01268600	0.71373000
O	2.73542200	1.27075000	-0.72959900
H	-5.30021100	1.01787500	1.04834300
H	-3.31102900	2.46136500	0.97855200
H	-5.16734900	-1.34230800	0.32500300
H	0.37395400	-2.78153900	-1.14015200
H	-1.66452600	-4.15407400	-1.24493800
H	-3.84531300	-3.23763100	-0.50623300
H	-1.78226500	3.66983800	0.28499700
H	0.25030100	4.98756400	-0.11164400
H	2.42225500	3.83089600	-0.57656500
H	5.02707100	0.27307000	-0.16126400
H	5.43137200	-1.98024900	0.84858300
H	3.48276000	-3.40657300	1.45253600
H	1.19576300	-2.64029100	0.99348500

Energies (0K) = -975.395452
Energies (0K) + ZPE = -975.379682
Enthalpies (298K) = -975.378738
Free Energies (298K) = -975.437774

Cation radical

C	-4.39124200	0.50635000	0.62920300
C	-3.29127800	1.35729500	0.60875100
C	-2.02476600	0.88626800	0.26029100
C	-1.86455800	-0.48635700	-0.03254400
C	-2.99700800	-1.34351300	-0.06883000
C	-4.25659600	-0.82129800	0.27766100
C	-0.58431300	-1.04147000	-0.29836700
C	-0.47365900	-2.36340500	-0.74723100
C	-1.59940000	-3.17084900	-0.83147100
C	-2.83978500	-2.68870100	-0.46388000
C	-0.87388100	1.74969000	0.10081800
N	0.52568700	-0.23676700	-0.12670100
C	-0.94073100	3.14635200	0.09603000
C	0.19033100	3.90861900	-0.11667000
C	1.42752100	3.30968900	-0.34117600
C	1.51794700	1.93829700	-0.33416600
C	0.38229700	1.14527500	-0.10818300
C	1.82884000	-0.74096000	0.11714800
C	2.91242800	0.09089500	-0.17689600
C	4.21461900	-0.34581400	-0.00928500
C	4.44410200	-1.60596900	0.51349300
C	3.37387000	-2.40849900	0.90174800
C	2.07453300	-1.97862000	0.71466700
O	2.71870600	1.36358700	-0.61492500
H	-5.36574300	0.89936100	0.90582400
H	-3.43282100	2.40417700	0.86079500
H	-5.12022000	-1.48292600	0.26411000
H	0.48812200	-2.75177400	-1.06461000
H	-1.49397300	-4.18965100	-1.19307600
H	-3.71752500	-3.33037500	-0.50075900
H	-1.89520100	3.64355100	0.23626000
H	0.11552900	4.99238700	-0.12329200
H	2.32089800	3.89699600	-0.53440400
H	5.02858100	0.32526400	-0.27000400
H	5.46477700	-1.95095800	0.65445800
H	3.55377800	-3.37262900	1.36867700
H	1.24871100	-2.59438900	1.05566200

Energies (0K) = -975.212530

Energies (0K) + ZPE = -975.196845
Enthalpies (298K) = -975.195900
Free Energies (298K) = -975.255329

triplet state

C	-4.38806000	0.55929700	0.65807900
C	-3.30199900	1.38067200	0.67714900
C	-1.99296300	0.89032700	0.30463600
C	-1.85215200	-0.49955100	-0.00980100
C	-3.01105600	-1.33593700	-0.08436600
C	-4.27010300	-0.79618000	0.26051500
C	-0.60147300	-1.06147400	-0.28782900
C	-0.48817700	-2.39460700	-0.77050900
C	-1.60981400	-3.17672700	-0.87060800
C	-2.86231600	-2.66692400	-0.50029300
C	-0.87144400	1.73504400	0.11653200
N	0.53134100	-0.25695600	-0.15193000
C	-0.91075900	3.15042300	0.14068200
C	0.21804600	3.90045600	-0.10670100
C	1.44636200	3.29843600	-0.39240400
C	1.50841200	1.91847600	-0.41637100
C	0.38732000	1.13802200	-0.16054700
C	1.82323200	-0.74843300	0.12290000
C	2.90760400	0.07630500	-0.19413300
C	4.20824300	-0.33721400	0.02311400
C	4.44707800	-1.58210900	0.58916600
C	3.37890100	-2.38458500	0.97037200
C	2.07469700	-1.96648800	0.75422500
O	2.70390700	1.31781000	-0.73026400
H	-5.36298700	0.94448400	0.94968700
H	-3.41644500	2.41653100	0.98633700
H	-5.14534900	-1.44224600	0.23269000
H	0.47717200	-2.77389300	-1.09032000
H	-1.52831200	-4.19218900	-1.24989600
H	-3.74813100	-3.29682800	-0.56609900
H	-1.85241900	3.65589300	0.33447300
H	0.15304500	4.98605700	-0.08627900
H	2.34150900	3.87945700	-0.59573000
H	5.02058500	0.33174500	-0.25036800
H	5.46949300	-1.91111500	0.75606600
H	3.55760900	-3.34124100	1.45453000
H	1.24611600	-2.58380400	1.08770100

Energies (0K) = -975.325164
Energies (0K) + ZPE = -975.308982

Enthalpies (298K) = -975.308038
Free Energies (298K) = -975.368714

PSH

Singlet state

C	-4.38806000	0.55929700	0.65807900
C	-3.30199900	1.38067200	0.67714900
C	-1.99296300	0.89032700	0.30463600
C	-1.85215200	-0.49955100	-0.00980100
C	-3.01105600	-1.33593700	-0.08436600
C	-4.27010300	-0.79618000	0.26051500
C	-0.60147300	-1.06147400	-0.28782900
C	-0.48817700	-2.39460700	-0.77050900
C	-1.60981400	-3.17672700	-0.87060800
C	-2.86231600	-2.66692400	-0.50029300
C	-0.87144400	1.73504400	0.11653200
N	0.53134100	-0.25695600	-0.15193000
C	-0.91075900	3.15042300	0.14068200
C	0.21804600	3.90045600	-0.10670100
C	1.44636200	3.29843600	-0.39240400
C	1.50841200	1.91847600	-0.41637100
C	0.38732000	1.13802200	-0.16054700
C	1.82323200	-0.74843300	0.12290000
C	2.90760400	0.07630500	-0.19413300
C	4.20824300	-0.33721400	0.02311400
C	4.44707800	-1.58210900	0.58916600
C	3.37890100	-2.38458500	0.97037200
C	2.07469700	-1.96648800	0.75422500
O	2.70390700	1.31781000	-0.73026400
H	-5.36298700	0.94448400	0.94968700
H	-3.41644500	2.41653100	0.98633700
H	-5.14534900	-1.44224600	0.23269000
H	0.47717200	-2.77389300	-1.09032000
H	-1.52831200	-4.19218900	-1.24989600
H	-3.74813100	-3.29682800	-0.56609900
H	-1.85241900	3.65589300	0.33447300
H	0.15304500	4.98605700	-0.08627900
H	2.34150900	3.87945700	-0.59573000
H	5.02058500	0.33174500	-0.25036800
H	5.46949300	-1.91111500	0.75606600
H	3.55760900	-3.34124100	1.45453000
H	1.24611600	-2.58380400	1.08770100

Energies (0K) = -1298.375380

Energies (0K) + ZPE = -1298.358985
Enthalpies (298K) = -1298.358041
Free Energies (298K) = -1298.418620

Cation radical

C	-4.49596100	0.54303800	0.64084400
C	-3.37725700	1.36472500	0.70871100
C	-2.11684800	0.89534300	0.32917200
C	-1.98984300	-0.44908000	-0.08249200
C	-3.13915100	-1.27670200	-0.19631300
C	-4.38959500	-0.75418500	0.17771000
C	-0.71917900	-1.00553100	-0.36804000
C	-0.62203100	-2.30648300	-0.88042700
C	-1.76003400	-3.08341800	-1.03876900
C	-3.00117800	-2.59528600	-0.67694600
C	-0.94724200	1.74113500	0.23552700
N	0.41076500	-0.24036100	-0.14103100
C	-1.00526500	3.13492800	0.35835500
C	0.11660000	3.91220900	0.17598800
C	1.33245700	3.32656600	-0.17062200
C	1.42740900	1.95513100	-0.30109000
C	0.30059700	1.14707100	-0.06171900
C	1.64516500	-0.86735200	0.20544600
C	2.86384200	-0.28542500	-0.14986900
C	4.05874200	-0.92220600	0.17006200
C	4.04339100	-2.11845400	0.86528100
C	2.83404200	-2.66089900	1.28856600
C	1.64390700	-2.03555800	0.97160900
S	2.90885100	1.26993300	-0.97623900
H	-5.46441100	0.93430700	0.94015100
H	-3.49916700	2.38742100	1.05314200
H	-5.26886900	-1.39046300	0.10328300
H	0.34500800	-2.69749700	-1.17994100
H	-1.66548900	-4.08343600	-1.45210400
H	-3.89066800	-3.21378100	-0.77530800
H	-1.95249400	3.61702500	0.57720900
H	0.05116100	4.99188100	0.27781600
H	2.20554900	3.94683600	-0.35944500
H	5.00287200	-0.46151700	-0.11212300
H	4.98086100	-2.60964800	1.11233100
H	2.81818700	-3.56896700	1.88469100
H	0.70509600	-2.44595600	1.33143300

Energies (0K) = -1298.189743
Energies (0K) + ZPE = -1298.173353

Enthalpies (298K) = -1298.172409
Free Energies (298K) = -1298.233564

triplet state

C	-4.46491100	0.62108800	0.72678600
C	-3.35402200	1.40300800	0.81410200
C	-2.06311200	0.90898000	0.37933300
C	-1.97426000	-0.44808500	-0.06545300
C	-3.15774700	-1.24095300	-0.20243000
C	-4.39564600	-0.69479100	0.20311500
C	-0.74374900	-1.01516700	-0.39643500
C	-0.66446600	-2.31713300	-0.96501700
C	-1.80724800	-3.05591400	-1.12629000
C	-3.04813500	-2.53631200	-0.72566800
C	-0.91908800	1.73149100	0.24291400
N	0.42065900	-0.26185700	-0.21106900
C	-0.93340900	3.13998600	0.40500300
C	0.19214000	3.89992400	0.18076900
C	1.38707000	3.30957000	-0.23408600
C	1.43452500	1.93011900	-0.40441900
C	0.32044500	1.13947600	-0.13811500
C	1.62401600	-0.88684000	0.20389800
C	2.85687400	-0.31441600	-0.12058300
C	4.04017500	-0.91664900	0.29004800
C	4.00627400	-2.09716800	1.01720100
C	2.78260100	-2.64851800	1.37775100
C	1.60003300	-2.03947800	0.99137800
S	2.90160200	1.17997900	-1.07380300
H	-5.42494000	1.00783300	1.06239600
H	-3.43597200	2.40726600	1.22115200
H	-5.29216400	-1.30708700	0.12888300
H	0.29773400	-2.70321100	-1.28912400
H	-1.75356600	-4.04541900	-1.57314300
H	-3.95163500	-3.13308200	-0.84097900
H	-1.85735800	3.63359600	0.69176100
H	0.14834000	4.97841700	0.31616200
H	2.27013100	3.91295200	-0.42979400
H	4.99141800	-0.45349900	0.03547500
H	4.93552600	-2.57093000	1.32371000
H	2.74510900	-3.55148200	1.98208400
H	0.64697400	-2.45532800	1.30694200

Energies (0K) = -1298.305221
Energies (0K) + ZPE = -1298.288367
Enthalpies (298K) = -1298.287423

Free Energies (298K) = -1298.349807

PCH

Singlet state

C	4.57048200	1.05442400	-0.66614800
C	3.33285000	1.70853400	-0.76526100
C	2.16459500	1.07962700	-0.38207900
C	2.22648700	-0.26885700	0.06780400
C	3.48576300	-0.91356900	0.22198600
C	4.65356300	-0.21653000	-0.16281200
C	1.04130600	-0.99201000	0.37155200
C	1.12936500	-2.25188900	0.92834700
C	2.37970200	-2.86010200	1.11479500
C	3.53595000	-2.22313500	0.74974400
C	0.86644100	1.75492900	-0.31662500
N	-0.19486500	-0.39261000	0.09159500
C	0.72006000	3.13451100	-0.48177600
C	-0.51708000	3.73721500	-0.36666500
C	-1.63406400	2.97800400	-0.03460900
C	-1.52946300	1.60957200	0.16885800
C	-0.27960300	1.00327200	-0.01910900
C	-1.33447400	-1.13381600	-0.31181000
C	-2.59833200	-0.57342500	-0.08902400
C	-3.71790700	-1.25216600	-0.56192000
C	-3.60101600	-2.46229500	-1.22951600
C	-2.34162800	-2.99991200	-1.45533900
C	-1.21224400	-2.33526800	-1.00907900
C	-2.66643100	0.73624300	0.67598400
C	-2.42899800	0.44536900	2.16889100
C	-4.02006600	1.41662200	0.54701300
H	5.47292700	1.57855900	-0.97332400
H	3.31104700	2.73037000	-1.13469300
H	5.61718400	-0.71095700	-0.05147800
H	0.22464900	-2.78491600	1.20752700
H	2.41842900	-3.85747100	1.54736500
H	4.50419500	-2.70621900	0.86708800
H	1.59128500	3.74766700	-0.69725200
H	-0.61415100	4.81070200	-0.50962100
H	-2.59233700	3.47506700	0.08882000
H	-4.70760100	-0.83152600	-0.40517200
H	-4.49175200	-2.97834300	-1.57990600
H	-2.23105300	-3.93595100	-1.99810300
H	-0.22826500	-2.74498600	-1.21805500

H	-3.22836400	-0.19793800	2.55789100
H	-2.43101700	1.38019100	2.74341600
H	-1.47372800	-0.05973300	2.34966300
H	-4.04686300	2.34144300	1.13263400
H	-4.26692400	1.66206500	-0.49285500
H	-4.81516500	0.77816000	0.94566500

Energies (0K) = -1017.976500

Energies (0K) + ZPE = -1017.957854

Enthalpies (298K) = -1017.956910

Free Energies (298K) = -1018.021376

Cation radical

C	4.60411600	1.02121300	-0.55528400
C	3.39470100	1.69228700	-0.67740700
C	2.18629900	1.06617800	-0.34782800
C	2.21560300	-0.28286900	0.06968400
C	3.45539900	-0.95697800	0.23579300
C	4.64395000	-0.27958100	-0.08856000
C	1.01471600	-0.99702000	0.30397400
C	1.06376700	-2.30056300	0.82291900
C	2.28361200	-2.92500200	1.03079100
C	3.46720200	-2.28221400	0.71634900
C	0.91585600	1.75058200	-0.32665700
N	-0.18604300	-0.37931200	0.03397400
C	0.78435500	3.13521400	-0.51224700
C	-0.44581800	3.73733800	-0.41308900
C	-1.58069200	2.98993700	-0.07671300
C	-1.50775800	1.62668000	0.11834700
C	-0.25553800	1.00862300	-0.06183600
C	-1.35645900	-1.11970100	-0.31412500
C	-2.60612000	-0.55146700	-0.05002600
C	-3.73601000	-1.25606100	-0.44918200
C	-3.63061200	-2.47591300	-1.10375800
C	-2.38075000	-3.00013200	-1.40195900
C	-1.24055600	-2.31708200	-1.01971400
C	-2.65503700	0.78751700	0.65570100
C	-2.41833700	0.55934100	2.16166000
C	-4.00052000	1.47837900	0.49548800
H	5.52706400	1.53257300	-0.81511700
H	3.39986900	2.72100100	-1.02483200
H	5.59297500	-0.79839200	0.02870100
H	0.14294800	-2.80871000	1.09130200
H	2.30164400	-3.92818900	1.44704700
H	4.42324500	-2.78245800	0.85442900

H	1.66064800	3.74220000	-0.71727400
H	-0.53742200	4.80984800	-0.56290600
H	-2.52539700	3.50889000	0.05490200
H	-4.72312900	-0.84623100	-0.25734100
H	-4.53142200	-3.00525300	-1.40435300
H	-2.28871700	-3.93230200	-1.95295500
H	-0.26328000	-2.69861000	-1.29960100
H	-3.22618800	-0.05659500	2.57432000
H	-2.41221000	1.51914100	2.69168000
H	-1.46942300	0.05182300	2.36861900
H	-4.01730500	2.42489400	1.04431600
H	-4.24231200	1.68263700	-0.55394100
H	-4.80150600	0.86667800	0.92126300

Energies (0K) = -1017.803068

Energies (0K) + ZPE = -1017.784490

Enthalpies (298K) = -1017.783545

Free Energies (298K) = -1017.848331

triplet state

C	4.59860200	1.04109800	-0.60313700
C	3.41350300	1.69174400	-0.75455900
C	2.16317300	1.05138100	-0.39774300
C	2.20314100	-0.31095000	0.03685800
C	3.45874000	-0.96512000	0.24448800
C	4.64825400	-0.27780800	-0.08501300
C	1.02646700	-1.01730000	0.28942300
C	1.06415400	-2.31756000	0.86700600
C	2.27193300	-2.92213300	1.09764800
C	3.46676900	-2.26632600	0.76396200
C	0.92619000	1.73643400	-0.32983700
N	-0.19632600	-0.39189900	0.03680800
C	0.77973400	3.13248400	-0.52067900
C	-0.44589200	3.73982500	-0.38040000
C	-1.57797000	3.00718800	-0.01445100
C	-1.49072100	1.63158400	0.18406900
C	-0.25871800	1.00997900	-0.02050700
C	-1.35347600	-1.10912100	-0.34112800
C	-2.60453900	-0.53157700	-0.08362900
C	-3.74022400	-1.20300600	-0.52317500
C	-3.65012900	-2.41646800	-1.19187400
C	-2.40367700	-2.96662300	-1.45637200
C	-1.25648000	-2.30996800	-1.04640900
C	-2.63994500	0.77320300	0.69126200
C	-2.39929700	0.46307500	2.17982400

C	-3.98219600	1.47875300	0.57612900
H	5.52664500	1.53664700	-0.88058200
H	3.40192700	2.70151200	-1.15643800
H	5.60254000	-0.78438000	0.04491900
H	0.13613200	-2.80822100	1.14576700
H	2.30486200	-3.91026900	1.54935000
H	4.42381400	-2.75730400	0.93325100
H	1.65018900	3.73428300	-0.76653100
H	-0.53317800	4.81310500	-0.53689100
H	-2.52327100	3.52396600	0.12073000
H	-4.72255700	-0.77591000	-0.34063000
H	-4.55494000	-2.92383500	-1.51785300
H	-2.31858600	-3.90079900	-2.00636100
H	-0.28050100	-2.71309400	-1.30139600
H	-3.20939500	-0.16633200	2.56960800
H	-2.37623600	1.39289700	2.76176800
H	-1.45366400	-0.06412800	2.34956300
H	-3.98586900	2.40135700	1.16554400
H	-4.23195800	1.73303000	-0.46084500
H	-4.78604600	0.85403600	0.97883100

Energies (0K) = -1017.905934
Energies (0K) + ZPE = -1017.886870
Enthalpies (298K) = -1017.885926
Free Energies (298K) = -1017.952027

PSeH

Singlet state

C	-4.66625800	0.95474000	0.73871600
C	-3.43634700	1.61965500	0.87091500
C	-2.26712600	1.04535500	0.41570600
C	-2.31686700	-0.26235800	-0.14218300
C	-3.56756100	-0.91607700	-0.32443100
C	-4.73931800	-0.27120100	0.13341200
C	-1.12781900	-0.93826500	-0.51774700
C	-1.19912800	-2.16206500	-1.14786100
C	-2.44065200	-2.77998200	-1.35927600
C	-3.60257200	-2.18601000	-0.94160900
C	-0.98096600	1.74827400	0.37354500
C	-0.87059400	3.12407700	0.59486000
C	0.34055100	3.78041900	0.47838400
C	1.47386100	3.07602200	0.09297900
C	1.37998400	1.71877200	-0.16427900
C	0.17370600	1.03719000	0.00501600

Se	2.89938700	0.75557800	-0.91090300
C	2.49271600	-0.83337200	0.13396400
C	1.15532800	-1.16803400	0.33464800
N	0.11494000	-0.35557600	-0.20324500
C	3.51201800	-1.60037100	0.68431800
C	3.20810100	-2.72747100	1.43028400
C	1.87670500	-3.05486600	1.65979900
C	0.86104900	-2.27506900	1.13382800
H	-5.57041100	1.43652600	1.10419500
H	-3.42381300	2.60695400	1.32501400
H	-5.69726200	-0.77080300	-0.00097800
H	-0.28495100	-2.66186000	-1.45816500
H	-2.46879600	-3.74882000	-1.85287400
H	-4.56423100	-2.67659100	-1.08035800
H	-1.75747100	3.69744100	0.85121900
H	0.40163200	4.84955100	0.66527900
H	2.42809800	3.58271600	-0.03307700
H	4.54678800	-1.30643500	0.52094000
H	4.00659200	-3.33680700	1.84630600
H	1.62279500	-3.91842700	2.26990400
H	-0.17555400	-2.53023500	1.33807500

Energies (0K) = -909.377768

Energies (0K) + ZPE = -909.360929

Enthalpies (298K) = -909.359984

Free Energies (298K) = -909.421996

Cation radical

C	-4.74489000	0.88935700	0.54947700
C	-3.55534000	1.58704600	0.71578700
C	-2.33001500	1.01477300	0.36006200
C	-2.31847400	-0.31132000	-0.12342400
C	-3.53689400	-1.01203100	-0.33163500
C	-4.74596200	-0.38449500	0.01480200
C	-1.09635700	-0.98066200	-0.37163100
C	-1.10176900	-2.26614000	-0.93294300
C	-2.30084300	-2.91449400	-1.18549300
C	-3.50528400	-2.31585000	-0.86729000
C	-1.07856900	1.73882200	0.36663800
C	-1.00676400	3.12297400	0.57211500
C	0.19114100	3.79382000	0.48516100
C	1.35624300	3.10888600	0.14313200
C	1.32426200	1.74692500	-0.06892100
C	0.12024500	1.03896100	0.08667800
Se	2.87775200	0.89361600	-0.84786100

C	2.52229200	-0.77375600	0.05605100
C	1.21247700	-1.14410300	0.35438900
N	0.09089800	-0.35028700	-0.04989700
C	3.58123400	-1.58352700	0.44780200
C	3.34305600	-2.74659400	1.15940300
C	2.04342300	-3.08160100	1.52396100
C	0.98665500	-2.28221700	1.13560800
H	-5.68287900	1.36078900	0.82910600
H	-3.59319600	2.59438500	1.11903100
H	-5.67934700	-0.92212100	-0.13648200
H	-0.16280300	-2.74418800	-1.19384900
H	-2.28459400	-3.90253900	-1.63585100
H	-4.44541100	-2.83554700	-1.03846900
H	-1.91286000	3.68398300	0.77652800
H	0.22682600	4.86680300	0.65141500
H	2.29197300	3.64741700	0.01574000
H	4.59799100	-1.28168400	0.20811000
H	4.17701800	-3.37392500	1.46269600
H	1.85138200	-3.96264500	2.12980300
H	-0.02313800	-2.53029200	1.45039500

Energies (0K) = 909.192768

Energies (0K) + ZPE = -909.176002

Enthalpies (298K) = -909.175057

Free Energies (298K) = -909.237473

triplet state

C	-4.71469600	0.92719300	0.63550800
C	-3.54408700	1.58963100	0.83162900
C	-2.28149100	1.00418200	0.42366800
C	-2.29586200	-0.32613800	-0.10309400
C	-3.53790400	-0.99263800	-0.34933100
C	-4.73951300	-0.35311300	0.02523100
C	-1.10732800	-0.98804800	-0.40070400
C	-1.11537900	-2.26448700	-1.03037800
C	-2.30903300	-2.88027900	-1.29591700
C	-3.51811700	-2.26249800	-0.93950300
C	-1.06253700	1.72044700	0.38950900
C	-0.95876200	3.11160500	0.64376100
C	0.23472800	3.78089300	0.50792200
C	1.38543200	3.11263800	0.08597900
C	1.31751800	1.74912700	-0.16968600
C	0.13736400	1.04067300	0.01936800
Se	2.85533400	0.82883200	-0.93082000

C	2.51910600	-0.76836900	0.12071400
C	1.19824700	-1.14053800	0.36912800
N	0.11156100	-0.36232700	-0.11471100
C	3.57108100	-1.52575100	0.61630700
C	3.31872300	-2.67285100	1.35333400
C	2.00667000	-3.03359900	1.63256400
C	0.95586100	-2.26539300	1.16204800
H	-5.65128400	1.38126200	0.95103200
H	-3.55327500	2.56464100	1.31083400
H	-5.68392100	-0.86926500	-0.13340500
H	-0.17332800	-2.72449600	-1.31449700
H	-2.32128900	-3.84827400	-1.78951900
H	-4.46407700	-2.76318800	-1.13822200
H	-1.84745000	3.66702500	0.92833600
H	0.28020500	4.84790600	0.71311500
H	2.32302700	3.64576900	-0.04766400
H	4.59370300	-1.21085900	0.42085500
H	4.14606900	-3.27046100	1.72678400
H	1.79670100	-3.91029600	2.23961000
H	-0.06749300	-2.52988800	1.41525200

Energies (0K) = -909.286020

Energies (0K) + ZPE = -909.268701

Enthalpies (298K) = -909.267757

Free Energies (298K) = -909.331636

Nap-PSeZ

Singlet state

C	3.84750300	-0.00073900	2.61607300
C	2.44434200	-0.00087600	2.75355600
C	1.63525500	-0.00054400	1.64902300
C	2.19395300	-0.00005500	0.34931400
C	3.61099500	0.00008400	0.20787100
C	4.41572900	-0.00027000	1.37122600
C	1.39537000	0.00029200	-0.82766700
C	1.98078100	0.00077200	-2.06758600
C	3.38302400	0.00090900	-2.20048100
C	4.17839700	0.00057100	-1.08740100
C	-0.20827100	2.33315500	-1.37954500
C	-0.87292700	3.55018500	-1.37662600
C	-2.06639400	3.70211900	-0.68605800
C	-2.57636200	2.62388300	0.02220700
C	-1.89162100	1.41761100	0.05374700
C	-0.70614400	1.23852900	-0.66639300

Se	-2.52255100	-0.00028200	1.22282200
C	-1.89148200	-1.41771700	0.05326000
C	-0.70602100	-1.23828200	-0.66682300
N	-0.02600900	0.00016300	-0.67085100
C	-2.57611500	-2.62404000	0.02131800
C	-2.06605300	-3.70199600	-0.68730300
C	-0.87260100	-3.54972500	-1.37781800
C	-0.20805500	-2.33263400	-1.38033700
H	4.47583700	-0.00101000	3.50386300
H	2.00251600	-0.00125300	3.74744300
H	0.55197100	-0.00065700	1.75894600
H	5.49844500	-0.00016100	1.25403000
H	1.34636200	0.00105700	-2.95235700
H	3.82580600	0.00129100	-3.19348000
H	5.26329400	0.00067700	-1.18148300
H	0.71590900	2.23344300	-1.94129500
H	-0.45253400	4.38390600	-1.93433100
H	-2.59641800	4.65121600	-0.69268400
H	-3.50523100	2.72058500	0.58150500
H	-3.50497400	-2.72101100	0.58058700
H	-2.59599200	-4.65113900	-0.69424200
H	-0.45213300	-4.38322500	-1.93579800
H	0.71611600	-2.23266500	-1.94205200

Energies (0K) = -910.536859
Energies (0K) + ZPE = -910.518661
Enthalpies (298K) = -910.517717
Free Energies (298K) = -910.584217

Cation radical

C	4.41437800	0.00031800	2.12425700
C	3.06653100	0.00047000	2.53721400
C	2.05389500	0.00029000	1.61571900
C	2.34756600	-0.00005800	0.23266100
C	3.70785700	-0.00020700	-0.18911300
C	4.72603800	-0.00001000	0.79237600
C	1.35503500	-0.00026800	-0.77753200
C	1.66723100	-0.00059000	-2.10773900
C	3.01529500	-0.00073600	-2.51087100
C	4.00951100	-0.00054800	-1.57083400
C	0.14145300	2.40988400	-0.38405400
C	-0.40619700	3.66016500	-0.23813200
C	-1.76471600	3.82085100	0.05247900
C	-2.55432600	2.70631300	0.19454600
C	-2.01263000	1.42534100	0.05195200

C	-0.64427200	1.24568200	-0.24477600
Se	-3.24126000	0.00024300	0.26125600
C	-2.01291200	-1.42512400	0.05212600
C	-0.64449400	-1.24577400	-0.24451200
N	-0.04466600	-0.00011400	-0.40078100
C	-2.55487200	-2.70597000	0.19485100
C	-1.76546300	-3.82068200	0.05304700
C	-0.40687300	-3.66030200	-0.23740800
C	0.14102900	-2.41015000	-0.38348400
H	5.20484900	0.00046700	2.87075000
H	2.83102200	0.00073300	3.59874000
H	1.01742800	0.00041500	1.94682600
H	5.76371300	-0.00012600	0.46351800
H	0.86590400	-0.00073400	-2.84407400
H	3.25631900	-0.00099400	-3.57033500
H	5.05525100	-0.00065300	-1.87317300
H	1.19801200	2.32341400	-0.60997800
H	0.23298100	4.53137100	-0.35042600
H	-2.19237000	4.81265800	0.16575700
H	-3.61394200	2.80541800	0.42033600
H	-3.61453100	-2.80483600	0.42054500
H	-2.19332100	-4.81238900	0.16642200
H	0.23215900	-4.53164600	-0.34945700
H	1.19763000	-2.32392100	-0.60930300

Energies (0K) = -910.350986
Energies (0K) + ZPE = -910.332918
Enthalpies (298K) = -910.331974
Free Energies (298K) = -910.398496

triplet state

C	4.02205400	0.00038400	2.56959800
C	2.68030600	0.00048100	2.78872400
C	1.77501800	0.00019200	1.68265500
C	2.24276900	-0.00017000	0.36688600
C	3.66544100	-0.00028200	0.13145200
C	4.52415600	-0.00000400	1.23278200
C	1.37956200	-0.00046700	-0.75537400
C	1.90689600	-0.00086400	-2.10765400
C	3.24339600	-0.00095400	-2.31190600
C	4.14362000	-0.00068000	-1.19524800
C	-0.12685400	2.34185100	-1.21578200
C	-0.78031900	3.56416900	-1.25722800
C	-2.03288500	3.71591800	-0.68110600
C	-2.61348900	2.63235600	-0.03741400

C	-1.94464600	1.41947800	0.03982900
C	-0.70068200	1.24010700	-0.57319500
Se	-2.67865400	0.00050400	1.14562200
C	-1.94513000	-1.41907200	0.04026300
C	-0.70112600	-1.24031100	-0.57285300
N	-0.02590200	-0.00022000	-0.53368400
C	-2.61439000	-2.63175300	-0.03657900
C	-2.03419200	-3.71569500	-0.67998700
C	-0.78160800	-3.56454200	-1.25622300
C	-0.12771000	-2.34244500	-1.21515300
H	4.72241900	0.00060500	3.40119000
H	2.28037800	0.00077400	3.79951300
H	0.70175500	0.00023500	1.86498500
H	5.59914800	-0.00008400	1.05962600
H	1.19909200	-0.00102300	-2.93438900
H	3.64877400	-0.00122400	-3.32069400
H	5.21837900	-0.00076400	-1.36639500
H	0.84787300	2.24640600	-1.68708800
H	-0.30172400	4.40262200	-1.75794500
H	-2.55334700	4.66933400	-0.72350300
H	-3.58806300	2.73062200	0.43741900
H	-3.58897700	-2.72954200	0.43833300
H	-2.55497500	-4.66895100	-0.72208400
H	-0.30333500	-4.40330200	-1.75673400
H	0.84704400	-2.24748600	-1.68650200

Energies (0K) = -910.446680

Energies (0K) + ZPE = -910.427773

Enthalpies (298K) = -910.426829

Free Energies (298K) = -910.496614

Nap-PXZ

Singlet state

C	3.99488800	-0.00000300	-2.08059700
C	2.64760100	-0.00000400	-2.49675400
C	1.63414100	-0.00000300	-1.57592800
C	1.92297000	-0.00000100	-0.19152900
C	3.28260300	0.00000000	0.23167400
C	4.30340700	0.00000000	-0.74759300
C	0.90992900	0.00000000	0.80406800
C	1.23042000	0.00000100	2.13552300
C	2.57714400	0.00000200	2.54725900
C	3.57850200	0.00000200	1.61492000
C	-0.51607100	-2.44747200	0.31897200

C	-1.22669300	-3.62381300	0.10219200
C	-2.56763500	-3.57930400	-0.23760800
C	-3.19820700	-2.34507700	-0.36107100
C	-2.49359100	-1.17884900	-0.14327300
C	-1.13716100	-1.20768600	0.20310900
O	-3.18367600	0.00000100	-0.26896700
C	-2.49359000	1.17885000	-0.14327500
C	-1.13716100	1.20768600	0.20310900
N	-0.46188200	0.00000000	0.41526900
C	-3.19820400	2.34507900	-0.36107500
C	-2.56763200	3.57930500	-0.23761300
C	-1.22669100	3.62381400	0.10219000
C	-0.51607000	2.44747300	0.31897300
H	4.78762500	-0.00000300	-2.82519200
H	2.41425300	-0.00000600	-3.55904600
H	0.59601600	-0.00000300	-1.90211700
H	5.34106300	0.00000100	-0.41725500
H	0.42726000	0.00000200	2.87011500
H	2.81315900	0.00000300	3.60848400
H	4.62277300	0.00000200	1.92369000
H	0.53669100	-2.49212600	0.58647900
H	-0.71537600	-4.57806600	0.20251700
H	-3.12925600	-4.49395300	-0.40812800
H	-4.25055100	-2.26594200	-0.62479000
H	-4.25054800	2.26594400	-0.62479600
H	-3.12925200	4.49395500	-0.40813600
H	-0.71537400	4.57806600	0.20251800
H	0.53669200	2.49212600	0.58648200

Energies (0K) = -976.546474
Energies (0K) + ZPE = -976.529084
Enthalpies (298K) = -976.528140
Free Energies (298K) = -976.593080

Cation radical

C	4.01502100	-0.00000400	-2.06655200
C	2.67430200	-0.00000700	-2.50188800
C	1.64617300	-0.00000500	-1.59763500
C	1.91898700	0.00000100	-0.21085900
C	3.27141000	0.00000400	0.23484200
C	4.30535600	0.00000100	-0.72968700
C	0.91317900	0.00000400	0.78503700
C	1.19714300	0.00000900	2.12195300
C	2.53878700	0.00001200	2.54618100

C	3.54794500	0.00000900	1.62183400
C	-0.50469000	-2.43919900	0.36285900
C	-1.22088100	-3.59520700	0.16284700
C	-2.57243800	-3.54941300	-0.20692500
C	-3.20807400	-2.33835100	-0.37838300
C	-2.49130900	-1.16884900	-0.18039000
C	-1.13255400	-1.19650900	0.19531900
O	-3.14212400	-0.00000400	-0.35875200
C	-2.49131300	1.16884300	-0.18039400
C	-1.13255800	1.19650800	0.19531500
N	-0.47186500	0.00000100	0.37875700
C	-3.20808200	2.33834200	-0.37838900
C	-2.57245000	3.54940700	-0.20693500
C	-1.22089200	3.59520600	0.16283600
C	-0.50469800	2.43920100	0.36285100
H	4.81760800	-0.00000600	-2.79989100
H	2.45641800	-0.00001000	-3.56708900
H	0.61599800	-0.00000700	-1.94752000
H	5.33751300	0.00000400	-0.38417500
H	0.38304500	0.00001100	2.84387500
H	2.76256200	0.00001600	3.60926100
H	4.58812900	0.00001100	1.94240800
H	0.54131800	-2.47829200	0.65016900
H	-0.73018900	-4.55504300	0.29358000
H	-3.12320600	-4.47272900	-0.36012700
H	-4.25360300	-2.26737400	-0.66418700
H	-4.25361000	2.26736100	-0.66419100
H	-3.12322100	4.47272100	-0.36013800
H	-0.73020300	4.55504500	0.29356600
H	0.54131100	2.47829900	0.65015900

Energies (0K) = -976.361830

Energies (0K) + ZPE = -976.344724

Enthalpies (298K) = -976.343780

Free Energies (298K) = -976.407661

triplet state

C	4.28090400	0.49203900	-1.68793400
C	3.04242000	0.82058400	-2.17297200
C	1.87992200	0.47648300	-1.44382300
C	1.96477100	-0.18004700	-0.21532300
C	3.26319600	-0.55402900	0.28002700
C	4.39125900	-0.20543100	-0.46424700
C	0.82723500	-0.54532800	0.56457000
C	0.96067000	-1.38140500	1.74187800

C	2.19659200	-1.71021800	2.19663900
C	3.35775800	-1.29371200	1.48970300
C	-1.13249300	-2.41035700	-0.30293800
C	-2.12644900	-3.33052400	-0.59696800
C	-3.44728000	-2.92085200	-0.71948100
C	-3.76699000	-1.57914200	-0.56716600
C	-2.77690300	-0.66272500	-0.26839900
C	-1.44491300	-1.06441400	-0.09950100
O	-3.15010200	0.64471700	-0.17429300
C	-2.18892100	1.58151900	0.07816100
C	-0.84951100	1.23855900	0.29073200
N	-0.46448400	-0.11451600	0.21503000
C	-2.61357800	2.89697100	0.15287800
C	-1.70895800	3.90129500	0.45653600
C	-0.38202900	3.57304000	0.70380000
C	0.04175300	2.25592000	0.62818900
H	5.18033700	0.75692100	-2.23919900
H	2.94010000	1.35030900	-3.11744000
H	0.90271700	0.72301500	-1.85383400
H	5.37276000	-0.49345300	-0.08969400
H	0.06057000	-1.66167600	2.28395500
H	2.30716700	-2.28838200	3.11123400
H	4.34550400	-1.56974200	1.85471400
H	-0.09563400	-2.72852300	-0.24167800
H	-1.85881800	-4.37301700	-0.74728300
H	-4.22890400	-3.63833600	-0.95402700
H	-4.78526000	-1.21632700	-0.68406100
H	-3.66642700	3.10601000	-0.02064700
H	-2.04422500	4.93324700	0.51416100
H	0.33487600	4.34670900	0.96575600
H	1.07671400	2.00775300	0.84102600

Energies (0K) = -976.457247

Energies (0K) + ZPE = -976.439495

Enthalpies (298K) = -976.438551

Free Energies (298K) = -976.504306
